# Calreticulin enhances the secretory trafficking of a misfolded α-1-antitrypsin

**DOI:** 10.1074/jbc.RA120.014372

**Published:** 2021-01-13

**Authors:** Harihar Milaganur Mohan, Boning Yang, Nicole A. Dean, Malini Raghavan

**Affiliations:** Department of Microbiology and Immunology, Michigan Medicine, University of Michigan, Ann Arbor, Michigan, 48109 USA

**Keywords:** calreticulin, calnexin, α1-antitrypsin, COPII, secretory trafficking, glycoprotein secretion, intracellular trafficking, chaperone, endoplasmic reticulum (ER), mass spectrometry (MS)

## Abstract

α1-antitrypsin (AAT) regulates the activity of multiple proteases in the lungs and liver. A mutant of AAT (E342K) called ATZ forms polymers that are present at only low levels in the serum and induce intracellular protein inclusions, causing lung emphysema and liver cirrhosis. An understanding of factors that can reduce the intracellular accumulation of ATZ is of great interest. We now show that calreticulin (CRT), an endoplasmic reticulum (ER) glycoprotein chaperone, promotes the secretory trafficking of ATZ, enhancing the media:cell ratio. This effect is more pronounced for ATZ than with AAT and is only partially dependent on the glycan-binding site of CRT, which is generally relevant to substrate recruitment and folding by CRT. The CRT-related chaperone calnexin does not enhance ATZ secretory trafficking, despite the higher cellular abundance of calnexin-ATZ complexes. CRT deficiency alters the distributions of ATZ-ER chaperone complexes, increasing ATZ-BiP binding and inclusion body formation and reducing ATZ interactions with components required for ER-Golgi trafficking, coincident with reduced levels of the protein transport protein Sec31A in CRT-deficient cells. These findings indicate a novel role for CRT in promoting the secretory trafficking of a protein that forms polymers and large intracellular inclusions. Inefficient secretory trafficking of ATZ in the absence of CRT is coincident with enhanced accumulation of ER-derived ATZ inclusion bodies. Further understanding of the factors that control the secretory trafficking of ATZ and their regulation by CRT could lead to new therapies for lung and liver diseases linked to AAT deficiency.

The endoplasmic reticulum (ER) is responsible for ensuring that protein quality control is maintained throughout various complex steps that allow proteins to reach their native state after being synthesized as an unstructured polypeptide chain (1). Crucial to the function of the ER are molecular chaperones that fall into several classical chaperone families such as the heat shock protein (HSP) family, which includes HSP47 and HSP70s (to which binding immunoglobulin protein, BiP, belongs) (2). Another group of chaperones that is particularly important for the folding of *N*-linked glycosylated proteins includes the lectin chaperones calreticulin (CRT), calnexin (CNX), and calmegin (CLGN) (2, 3, 4). Whereas CRT is soluble, CNX and CLGN are type I transmembrane proteins. The ER lumenal domains of these chaperones are structurally related, containing a globular domain that is responsible for the binding of nascent monoglucosylated glycoproteins and a P/arm-domain responsible for the recruitment of co-chaperones, including the thiol-oxidoreductase ERp57 (5, 6, 7) (Fig. 1*A*). Unique to CRT is an acidic C-terminal region, which contains multiple low-affinity calcium-binding sites (8, 9, 10) (Fig. 1*A*). Calcium is a key ionic component of the ER known to be important for proper protein folding and secretion. Calcium concentrations in the ER range from 100–800 μm, compared with 100 nm in the cytosol and 1–2 mm in the extracellular space (11). Through its low-affinity calcium-binding sites, CRT plays a central role in ER calcium storage and cellular calcium homeostasis (12).

**Figure 1:**
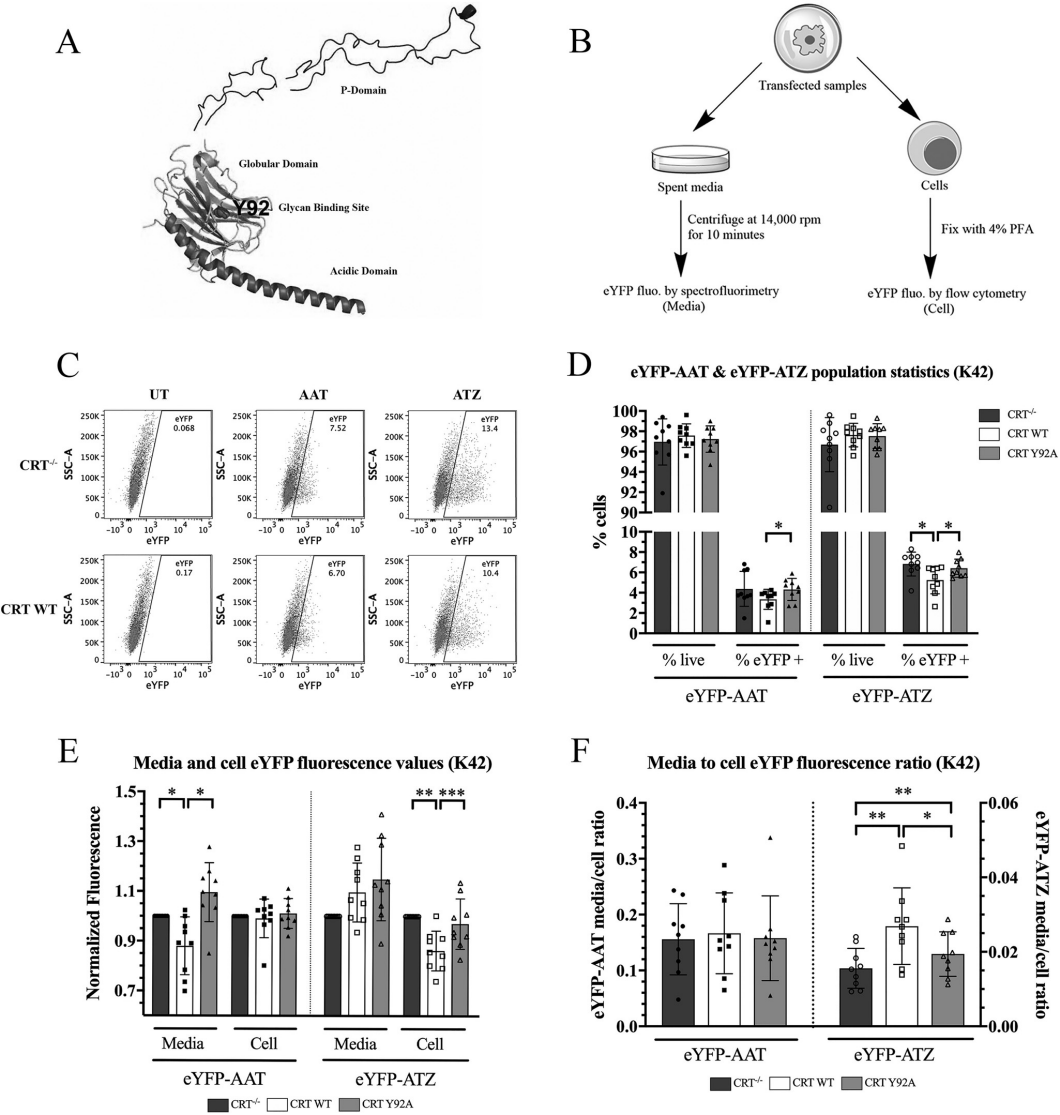
**CRT enhances ATZ trafficking in K42 mouse embryonic fibroblasts, and this effect is partially dependent on glycan binding.***A*, EM structure of CRT (PDB ID 6ENY) depicting the glycan-binding site which includes residue Tyr-92 within the globular domain. CRT's acidic domain is helical and the P-domain forms an *extended* β-hairpin structure. *B*, workflow for the estimation of media and cellular fluorescence in Fig. 1, Fig. 3, Fig. 4, Fig. S1, and Fig. S3. Briefly, media was collected 48 h post-transfection and cleared of debris by high-speed centrifugation. eYFP fluorescence was measured at an excitation wavelength of 514 nm and emission wavelength of 527 nm. Cellular fluorescence (post-fixation) was measured in the FITC channel on a flow cytometer. *C*, representative flow cytometry dot plots of cellular eYFP fluorescence in untransfected, eYFP-AAT–transfected, or eYFP-ATZ–transfected K42 CRT^−/−^ and CRT WT or CRT Y92A cells. *D*, percentage live cells of all cells (pre-gated on forward and side scatter) and percentage of eYFP^+^ cells identified from the total live cell population. *E*, total media fluorescence and cell MFI values were normalized relative to corresponding values from CRT^−/−^ cells in eYFP-AAT– or eYFP-ATZ–transfected cells. *F*, the ratio between the media and cell fluorescence values calculated as $\frac{Mediafluorescence}{CellulareYFPMFI\times Numberof{eYFP}^{+}cells}$. For *D*–*F*, data were obtained from nine independent transfections of the indicated K42 cells and are shown as mean ± S.D. (*error bars*). Repeated measures (RM) one-way ANOVA analysis was performed for each set of measurements, comparing CRT^−/−^, CRT WT, and CRT Y92A. Because the data in *panel E* is normalized, for this panel, RM one-way ANOVA analysis was performed on the log-transformed data. Only significant comparisons are indicated. *p < 0.05, **p < 0.01, ***p < 0.001. See also Fig. S1 for additional replicates comparing K42 CRT^−/−^ and CRT WT cells and Fig. S3, A–C for individual experimental trends of the eYFP-ATZ data.

α1-antitrypsin (AAT) is a 52-kDa glycoprotein, a highly abundant serum serine protease inhibitor (serpin) that binds to and irreversibly inhibits members of the chymotrypsin family, serine proteases, including but not limited to neutrophil elastase, via a molecular “mouse-trap” mechanism (13). AAT has several genetic variants, which fall into three main categories: normal alleles (such as M1, M2, M3, and M4), deficient alleles characterized by missense mutations (for example, the Z and S variants), and null/truncation mutants that have a stop codon insertion (the null Hong Kong (NHK) and Saar variants) (14). Expression of many of the deficient and null alleles results in misfolding and/or polymerization of their protein products (15). Of these, the Z mutation characterized by a Glu-to-Lys substitution at position 342 (E342K) in the mature protein is particularly debilitating (14). The Z allele encoding α1-antitrypsin Z (ATZ) is autosomal codominant with a frequency of 2–5% in Caucasians of European ancestry (14). ATZ is misfolded and forms polymers or aggregates within the cell (16, 17). Polymerization also leads to reduced secretion of the protein into the serum, leading to the disease α1-antitrypsin deficiency (ATD), resulting in proteolytic damage of lung tissue and lung emphysema. The other highly studied phenotype is the accumulation of aggregated ATZ within hepatocytes resulting in cellular toxicity that, in some people, leads to liver cirrhosis and hepatocellular carcinoma (16). Aggregated ATZ is a very well-characterized target for much of the cell's degradative machinery, including ER-associated degradation (ERAD) (18, 19), autophagy (18, 20, 21), and ER-to-lysosome-associated degradation (ERLAD) (22).

Various studies have demonstrated roles of CNX in folding, ER retention, cellular sequestration, secretory trafficking, and degradation of AAT and its mutants (22, 23, 24, 25, 26, 27, 28, 29, 30, 31, 32, 33), whereas CRT's functions within these pathways are not well-studied. Elucidation of factors that influence folding, cellular retention, degradation, and secretion of ATZ in hepatocytes is important for gaining a better understanding of variable susceptibilities to liver disease in patients homozygous for the Z allele (16). A recent study showed that UDP-glucose:glycoprotein glucosyltransferase 1 (UGGT1), the enzyme that regenerates monoglucosylated glycans on glycoproteins for CNX/CRT binding, can reduce insoluble NHK in a CRT-dependent manner (34). Based on this study, we hypothesized that CRT may also play a role in reducing ATZ accumulation within the cell by increasing its secretion and/or by promoting its degradation, both of which were investigated. Overall, our data provides a new and comprehensive view of CRT-specific roles in ATZ secretion and degradation.

## Results

### CRT enhances the secretory trafficking of ATZ via a mechanism that is partly dependent on its glycan-binding site

To examine cellular functions of CRT in ATZ folding and degradation, *Calr*^−/−^ mouse embryonic fibroblasts (MEFs, K42 cells (9)) were transduced with retroviruses that encoded either WT human CRT, the Y92A mutant of human CRT that is deficient for binding monoglucosylated glycans (35, 36) or a control virus lacking calreticulin (hereafter called CRT WT, CRT Y92A, or CRT^−/−^, respectively). The cells were then transfected with either enhanced YFP (eYFP)-AAT or eYFP-ATZ encoding plasmids (37). AAT/ATZ levels in the media and within cells were quantified at 48 h post-transfection, utilizing eYFP fluorescence as a readout (Fig. 1*B*). We estimated cellular fluorescence using flow cytometry, by gating on eYFP^+^ cells, using untransfected cells for the background control (Fig. 1*C*). In the case of eYFP-AAT, there were small differences in transfection efficiency (% eYFP^+^ cells) between the CRT WT and CRT^−/−^ cells and other conditions (Fig. 1*D* and Fig. S1A), which resulted in a parallel decrease in media fluorescence (Fig. 1*E* and Fig. S1B, *left panels*). However, in the case of eYFP-ATZ, we observed a small, nonsignificant increase in the media fluorescence of CRT WT over CRT^−/−^ cells, which was accompanied by a reduction in cellular eYFP mean fluorescence intensity (MFI) (Fig. 1*E* and Fig. S1B, *right panels*). Fewer eYFP^+^ cells yielded the increase the ATZ media fluorescence in the context of CRT WT (Fig. 1*D* and Fig. S1A, *right panels*), indicating that transfection differences did not account for the observed changes. Quantitative PCR revealed no differences in the levels of eYFP-ATZ transcripts between the K42 cells (Fig. S2A). In transfections with plasmids encoding either eYFP-AAT or eYFP-ATZ, we also did not detect significant differences in cell death (% live cells) from transfection (Fig. 1*D* and Fig. S1A). We next calculated the ratio between the secreted and intracellular eYFP fluorescence by dividing the media fluorescence (corrected for background by subtracting the media fluorescence of the corresponding untransfected cells) by the cell fluorescence (calculated as the product of cellular eYFP MFI and the number of eYFP^+^ cells) (Table S1). For eYFP-AAT, the presence of CRT did not affect the ratio (Fig. 1*F* and Fig. S1C, *left panels*). In contrast, CRT expression increased the media:cell fluorescence ratio for ATZ (Fig. 1*F* and Fig. S1C, *right panels*), indicating a role for CRT in altering the distribution of ATZ between media and cells.

CRT's ability to bind monoglucosylated glycoproteins is attributed to conserved residues in its globular lectin domain, of which Tyr-92 (Fig. 1*A*; PDB 6ENY (38)) is crucial because its substitution causes an impairment of its ability to bind substrates such as major histocompatibility complex 1 (MHC-1) molecules (35, 36). CRT WT and CRT Y92A were expressed at similar levels (Fig. S2B, *top panel*), which allowed further assessment of whether the ability of CRT to increase the secretory trafficking of eYFP-ATZ depended on glycan binding by CRT. In transfections with plasmids encoding eYFP-ATZ, the media fluorescence values were comparable between CRT WT and CRT Y92A, and both were slightly higher than that from CRT^−/−^ cells (Fig. 1*E*, *right panel*). In addition, there was a significant reduction in cell MFI of CRT WT compared with both CRT^−/−^ and CRT Y92A MEFs (Fig. 1*E*, *right panel*), along with fewer eYFP^+^ cells in the CRT WT condition (Fig. 1*D*, *right panel*). Interestingly, the significant increase in the eYFP-ATZ media:cell fluorescence ratio in the CRT WT condition compared with CRT^−/−^ was also measured for the CRT Y92A mutant. However, there was also a significant difference in the media:cell fluorescence ratio between CRT WT and the CRT Y92A mutant, indicating that compared with CRT WT, the CRT Y92A mutant is less efficient at altering eYFP-ATZ distribution (Fig. 1*F*, *right panel* for averaged data; see also individual experimental trends shown in Fig. S3, A–C). The statistical analyses in Fig. 1, Fig. S1, and Fig. S3 are based on paired two-tailed *t* tests (Fig. S1; *n* = 2 conditions being compared) or repeated measures (RM) one-way analysis of variance (ANOVA) analyses (Fig. 1 and Fig. S3, A–C, *n* = 3 conditions being compared). Trends within experiments (Fig. S3A) are compared for the statistical analyses of Fig. 1, because the flow cytometry settings and conditions of the experiments (such as numbers of cells analyzed and transfection reagents used) were kept consistent across the conditions being compared on a given day. On the other hand, neither CRT WT nor the CRT Y92A mutant altered the media:cell fluorescence ratio for eYFP-AAT (Fig. 1*F*, *left panel*). Thus, the glycan-binding deficient CRT Y92A mutant does not affect the trafficking of eYFP-AAT but improves eYFP-ATZ secretion, although to a lesser extent than CRT WT.

### CRT inhibits the in vitro insolubility of ATZ

**Figure 2:**
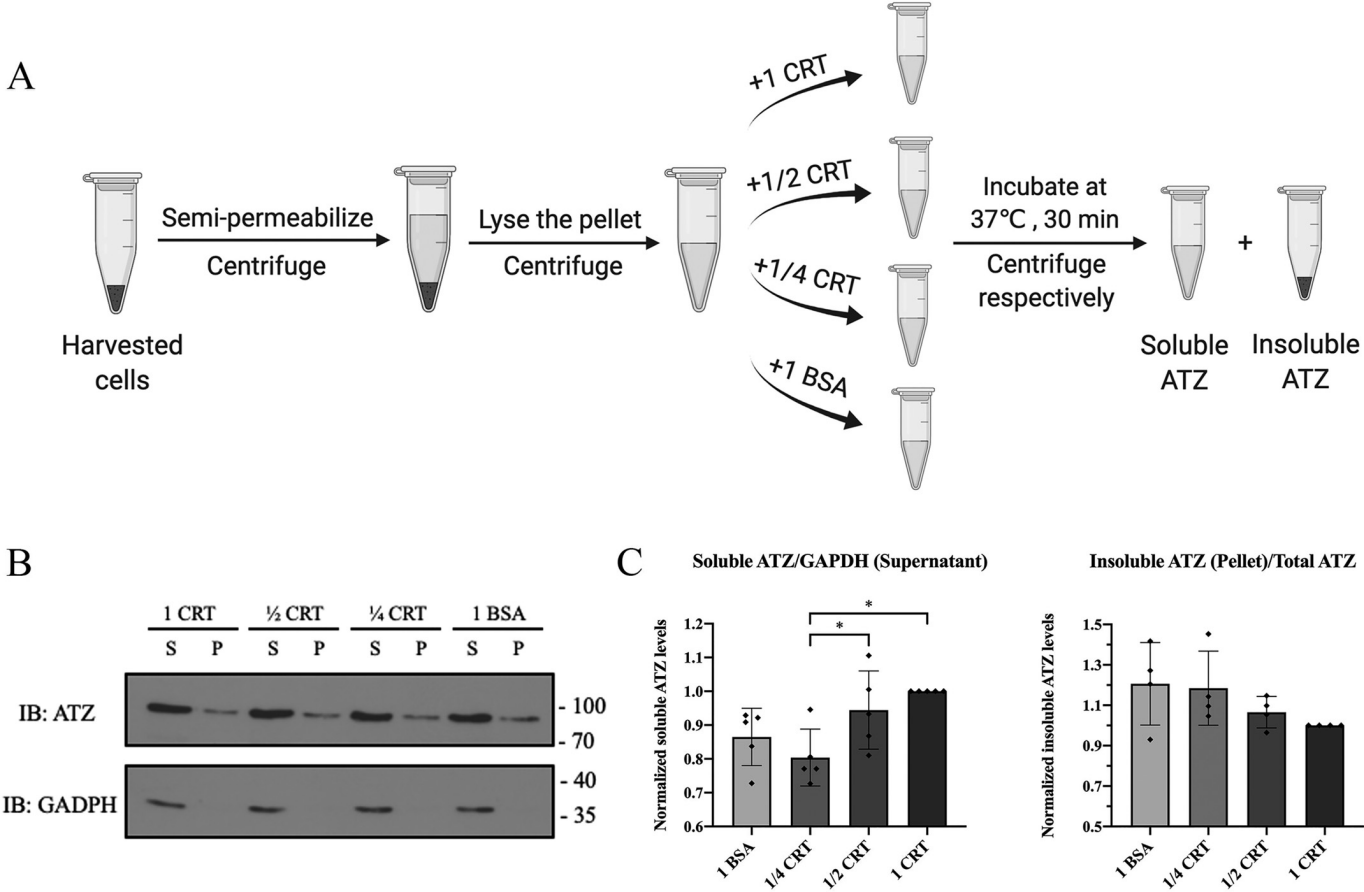
**CRT can reduce the insolubility of ATZ *in vitro*.***A* and *B*, workflow (*A*) and representative example (*B*) of the *in vitro* aggregation assay in eYFP-ATZ–transfected Huh7.5 CRT KO cells. After semi-permeabilization, the membrane-enriched fraction, which included the ER, was obtained and lysed in RIPA buffer containing 0.1% SDS. The ER-containing lysate was clarified by centrifugation and diluted 10-fold, and further incubated at 37 °C in the presence of purified recombinant calreticulin or BSA in equal microgram amounts relative to the total lysate protein content (1 CRT and 1 BSA, respectively), or one-half or one-quarter the amounts of calreticulin relative to the total lysate protein content. The samples were centrifuged, and the supernatant (*S*) and pellet (*P*) fractions (harboring soluble and insoluble eYFP-ATZ, respectively) were subject to SDS-PAGE and immunoblotting against eYFP-ATZ, using GAPDH as a loading control (*B*). *C*, data are quantified over at least four independent experiments and shown as mean ± S.D. (*error bars*). RM one-way ANOVA analysis was performed on log-transformed data to compare differences between the four conditions. *p < 0.05.

We further assessed whether human CRT is capable of preventing aggregation of eYFP-ATZ in an *in vitro* assay (Fig. 2*A*). *Calr* was knocked out using CRISPR/Cas9 gene editing in Huh7.5 cells, a human hepatocellular carcinoma-derived cell line (39) that secretes endogenous AAT (hereafter called WT or CRT KO, respectively, for the control Huh7.5 (transduced with an empty lentiviral vector) and CRISPR/Cas9-edited cells (transduced with sgRNA that targeted CRT)). The levels of CRT were below detection limits on immunoblots, thus confirming the knockout (Fig. S2B, *middle panel*). The Huh7.5 CRT KO cells were transfected with plasmids encoding eYFP-ATZ, semi-permeabilized with a low concentration of digitonin (0.01%), and centrifuged to generate a supernatant fraction (which consists of cytosolic components) and a pellet fraction enriched in cell membranes, including the ER. The pellet fraction was then lysed in RIPA buffer containing 0.1% SDS and further diluted 10-fold before incubation at 37 °C in the presence of purified recombinant human CRT or BSA. Insoluble eYFP-ATZ was pelleted by centrifugation and levels of eYFP-ATZ in both the supernatant and pellet fractions were quantified by immunoblotting. Although the effect is modest, we found that the presence of increasing amounts of human CRT contributed to a reduction in the levels of insoluble eYFP-ATZ (Fig. 2, *B* and *C*). Together, these findings of Fig. 2 support the model that direct interactions between CRT and ATZ can enhance ATZ folding and solubility, which at least in part can explain the ability of CRT to enhance the secretory trafficking of ATZ.

### In the hepatocyte cell line Huh7.5, CRT but not CNX enhances the secretory trafficking of ATZ

Various studies have shown roles for CNX in the binding, sequestration, and degradation of AAT and its mutants (22, 23, 24, 25, 26, 27, 28, 29, 30, 31, 32, 33). Because CNX and CRT are structurally related chaperones involved in glycoprotein folding, we compared CRT's role with that of CNX in influencing the media:cell fluorescence ratios of eYFP-AAT and eYFP-ATZ. For these analyses, we additionally knocked out *Canx* in the Huh7.5 cells using CRISPR/Cas9 gene editing (hereafter called WT or CNX KO, respectively, for the control Huh7.5 (transduced with an empty lentiviral vector) and CRISPR/Cas9-edited cells that targeted CNX (transduced with sgRNA that targeted CNX)). The levels of CNX were below detection limits on immunoblots, thus confirming the knockout (Fig. S2B, *bottom panel*). Media:cell fluorescence ratio measurements of eYFP-AAT and eYFP-ATZ were further assessed.

**Figure 3:**
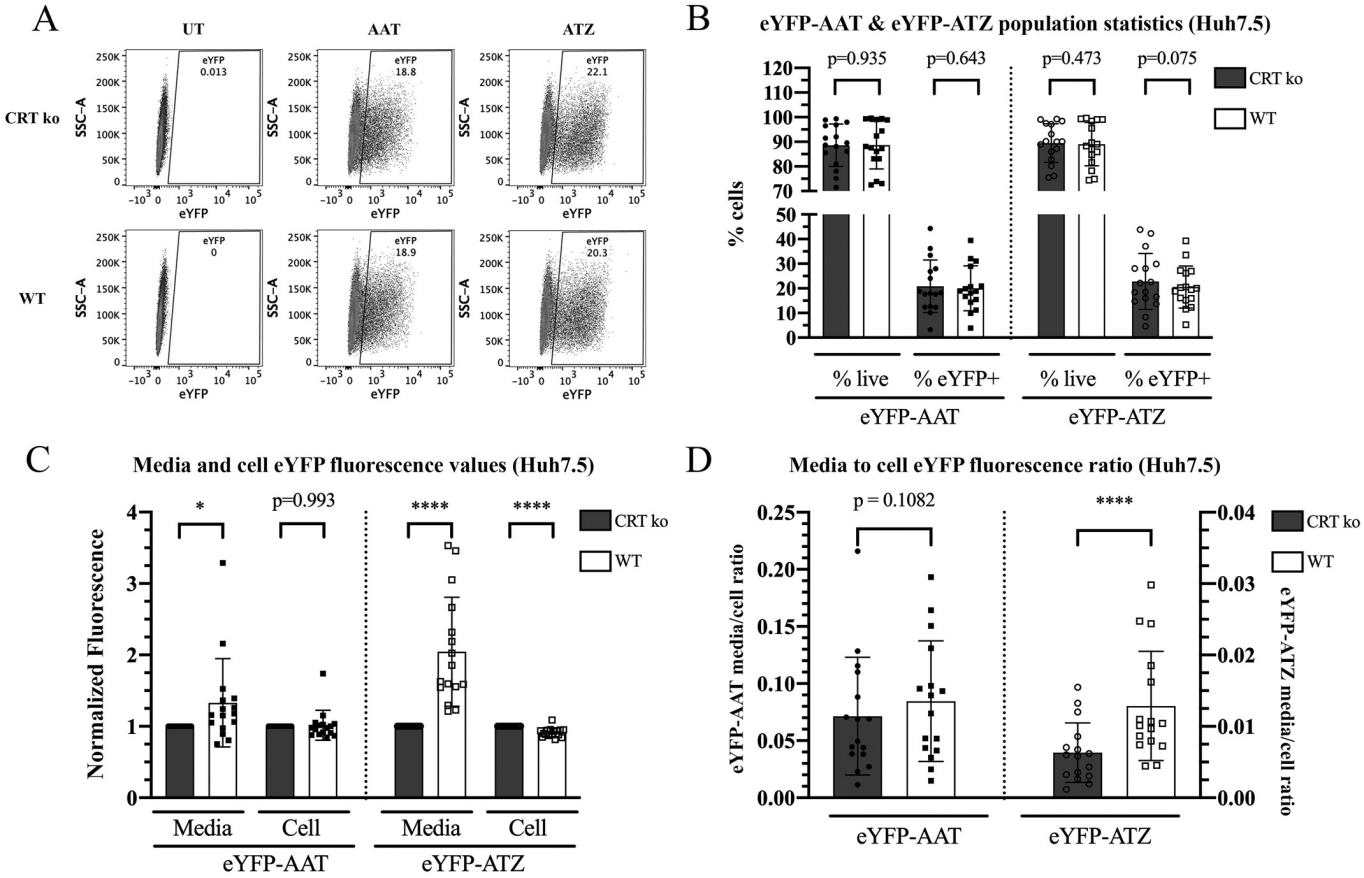
**CRT promotes the secretory trafficking of ATZ in Huh7.5 cells.***A*, representative flow cytometry dot plots of cellular eYFP fluorescence in untransfected, eYFP-AAT–transfected, or eYFP-ATZ–transfected Huh7.5 CRT-knockout (CRT KO) and WT (control vector) cells. *B*, total live cells expressed as a percentage of all cells (pre-gated on forward and side scatter), and percentage of eYFP^+^ cells identified from the total live cell population. *C*, total media fluorescence and cell MFI values were normalized relative to corresponding values from CRT KO cells in eYFP-AAT–transfected or eYFP-ATZ–transfected cells. *D*, the ratio between the media and cell fluorescence was obtained, as in Fig. 1. Data in (*B*–*D*) were obtained from 16 independent transfections of the Huh7.5 CRT KO and WT lines and are shown as mean ± S.D. Nine replicates were done in parallel with CNX data in Fig. 4. Paired two-tailed *t* tests were performed comparing CRT KO with WT conditions for each measurement. Because the data in *panel C* is normalized, for this panel, *t* tests were performed on log-transformed data. *p <0.05, ****p <0.0001. See also Fig. S3, D–F for individual experimental trends of the eYFP-ATZ data.

For analyses with the CRT KO cells, parallel to the procedures with K42 cells, the eYFP^+^ gates were set based on the background fluorescence of untransfected cells (Fig. 3*A*). There were no significant differences in cell viability (% live cells) or transfection efficiencies (% eYFP^+^ cells), although we still observed a slightly lower percentage of eYFP^+^ cells in the eYFP-ATZ transfected condition for WT cells compared with CRT KO, similar to that of WT K42 cells (Fig. 3*B*, *right panel* compared with Fig. 1*D*, right panel). This was also accompanied by elevated eYFP-ATZ media and reduced cellular fluorescence levels for WT (Fig. 3*C*, *right panel* for averaged data, and Fig. S3, D and E for individual replicate trends). Additionally, eYFP-AAT transfected WT cells had more eYFP fluorescence in the media compared with CRT KO cells (Fig. 3*C*, *left panel*). Finally, a very consistent and significant increase in the ratio of media:cellular eYFP-ATZ fluorescence was measured in WT compared with CRT KO Huh7.5 cells (Fig. 3*D*, *right panel* for averaged data and Fig. S3F for individual experiment trends; statistical analyses are based on paired two-tailed *t* tests). A smaller, nonsignificant increase in the media:cell fluorescence ratio was observed for eYFP-AAT (Fig. 3*D*, *left panel*). Therefore, in both K42 and Huh7.5 cell lines, compared with eYFP-AAT, CRT expression more specifically enhances the fraction of the total eYFP-ATZ protein pool that is secreted.

**Figure 4:**
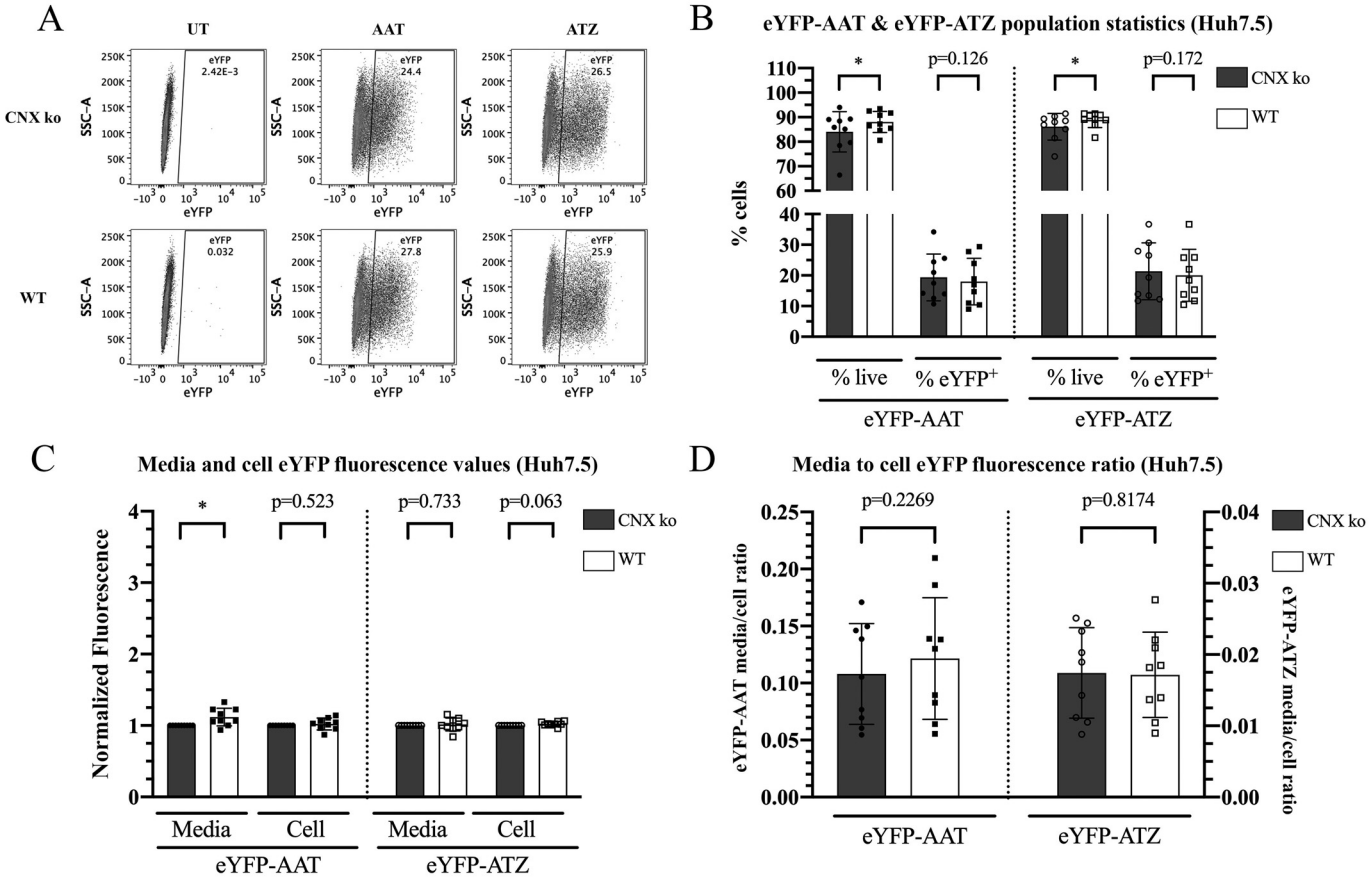
**CNX does not promote the secretory trafficking of ATZ in Huh7.5 cells.***A*, representative flow cytometry dot plots of cellular eYFP fluorescence in untransfected, eYFP-AAT–transfected, or eYFP-ATZ–transfected Huh7.5 CNX-knockout (CNX KO) and WT (control vector) cells. *B*, % live and % eYFP^+^ population statistics for eYFP-AAT– and eYFP-ATZ–transfected cells are shown (cells were pre-gated on forward and side scatter, live cells, then the eYFP^+^ population). *C*, total media fluorescence and cell MFI values were normalized relative to corresponding values from CNX KO cells in eYFP-AAT– or eYFP-ATZ–transfected cells. *D*, media:cell fluorescence ratios, calculated as described in Fig. 1. Data in *B*–*D* were obtained from nine independent transfections of the Huh7.5 CNX KO and WT lines and are shown as mean ± S.D. (*error bars*). Paired two-tailed *t* tests were performed comparing CNX KO with WT conditions for each measurement. Because the data in *panel C* is normalized, for this panel, *t* tests were performed on log-transformed data. *p <0.05, compared with CNX KO condition.

Next, Huh7.5 CNX KO or WT cells were transfected with either eYFP-AAT or eYFP-ATZ encoding plasmids and gated as described above (Fig. 4*A*). The small increase in media fluorescence of eYFP-AAT in WT cells compared with CNX KO cells may arise from the small parallel increase in cell viability (% live cells) in the presence of CNX (Fig. 4, *B* and *C*, *left panels*). Though the same trends in viability were present for eYFP-ATZ, we did not see significant differences in the media or cell fluorescence levels between CNX KO and WT cells. When the media:cell fluorescence ratios were compared, WT cells did not have an increased media:cell fluorescence ratio for either eYFP-AAT or eYFP-ATZ, indicative of fundamental differences in the roles of CRT *versus* CNX in secretory trafficking, particularly of eYFP-ATZ (Fig. 3*D* compared with Fig. 4*D*). Thus, in contrast to CRT, CNX does not positively influence eYFP-ATZ secretion in Huh7.5 cells.

### Small influences of CRT and CNX on ATZ degradation and polymeric ATZ accumulation

**Figure 5:**
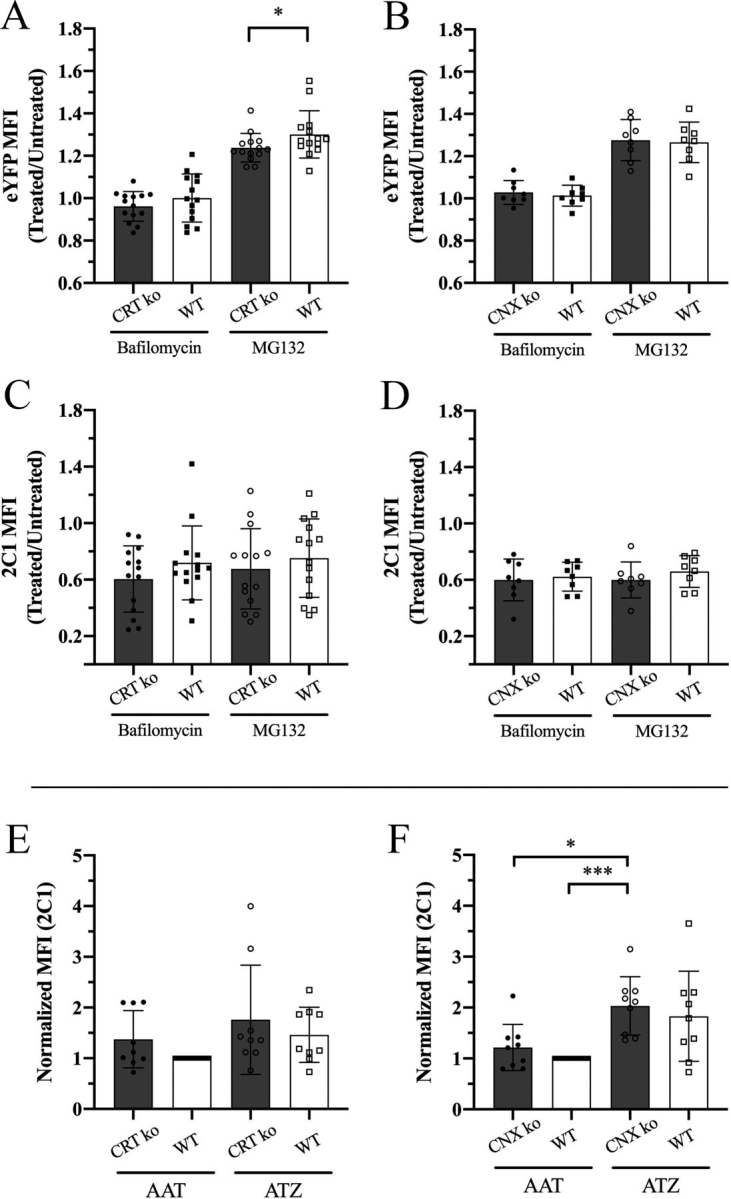
**Small influences of CRT and CNX on ATZ degradation and polymeric ATZ accumulation.***A*–*D*, Huh7.5 CRT KO, CNX KO, or corresponding WT cells as indicated were transfected with eYFP-ATZ–encoding plasmids and treated with either 100 nm bafilomycin or 10 µg/ml MG132 or left untreated at 20 h post-transfection. At 24 h post-transfection, the cells were harvested, stained with 2C1, and analyzed. The polymeric ATZ (2C1) gate was determined by gating on forward and side scatter, live cells, then eYFP^+^ cells. The secondary antibody staining control was used as the cutoff for setting the polymeric ATZ gate. For each cell type and drug-treatment condition, ratios of signals from drug-treated/untreated cells were measured to calculate eYFP MFI ratios (*A* and *B*) or 2C1 MFI ratios (*C* and *D*). Data for CRT KO and WT were obtained from 14 independent replicates, eight of which were conducted in parallel with CNX KO. Data for CNX KO and WT were obtained from eight independent replicates. All data are shown as mean ± S.D. *(error bars*). RM one-way ANOVA analysis was performed, and *p*-values are reported for comparisons of KO and WT conditions for each drug treatment. *E* and *F*, Huh7.5 CRT KO, CNX KO, or corresponding WT cells as indicated were transfected with eYFP-AAT– or eYFP-ATZ–encoding plasmids and stained with the polymer-selective antibody 2C1 at 48 h post-transfection. 2C1 MFI (of eYFP^+^ populations) is shown (gated as described in *A*–*D*). Data quantified over nine independent experiments, conducted in parallel, are shown as mean ± S.D. (*error bars*). Data are normalized relative to the WT eYFP-AAT transfected signals. RM one-way ANOVA analysis was performed on the log-transformed data in *E* and *F*. *p < 0.05, ***p < 0.001.

The findings that CRT-expressing cells had reduced intracellular eYFP-ATZ fluorescence and increased media:cell fluorescence ratios (Figure 1, Figure 2, Figure 3) might, in part, be explained by CRT-mediated enhancement of the degradation of ATZ. ATZ degradation is known to occur via ERAD, autophagy, and ERLAD (18, 19, 20, 21, 22). To further elucidate the influences of CRT in promoting eYFP-ATZ degradation via ERAD, we used MG132, an inhibitor of the catalytic activity of the 26S proteasome subunit (40). To examine CRT's role in any autophagy component-mediated pathway we used bafilomycin A1, a specific inhibitor of the lysosomal vacuolar H^+^-ATPase (41, 42). CRT KO, CNX KO (as controls), or corresponding WT cells were transfected with eYFP-ATZ. Drugs were added 20 h later, and flow cytometry analyses were used to quantify eYFP-ATZ and polymeric ATZ levels following inhibitor treatments or in untreated cells at 24 h post-transfection. Drug-treated signals as a ratio relative to untreated populations are shown in Fig. 5, *A*–*D* (MFI values) and Fig. S5, A–D (% cells).

When measuring eYFP signals, MG132 treatments increased eYFP-ATZ MFI values under all conditions (the treated/untreated ratios were above 1.0) (Fig. 5, *A* and *B*). In contrast, bafilomycin treatments resulted in small changes in eYFP MFI, suggesting a stronger role for proteasomal than lysosomal activity in eYFP-ATZ degradation in Huh7.5 cells. WT cells show a greater rescue of eYFP-ATZ MFI compared with CRT KO (but not CNX KO) cells following MG132-treatment, with the difference reaching a small significance when MFI values were compared (Fig. 5, *A* and *B* and Fig. S5, A and B). Similar results to those in Huh7.5 cells were obtained from K42 cells lacking or expressing CRT, where immunoblotting analyses were used to quantify the extent of eYFP-ATZ enrichment in the drug-treated cells compared with untreated cells (Fig. S4). Again, MG132 treatment induced stronger rescue of eYFP-ATZ compared with bafilomycin, particularly with WT cells. Overall, however, CRT appears to be nonessential for ATZ degradation, because the differences between CRT KO and WT cells are small or nonsignificant (Fig. 5*A*, Fig. S4, and Fig. S5A). Therefore, the increase in the media:cell fluorescence ratio of eYFP-ATZ when CRT is present is likely not due to a reduction in cellular ATZ via increased CRT-mediated degradation but rather due to an active role of CRT in mediating trafficking of ATZ out of the ER.

The antibody 2C1 is described as specific for polymeric forms of ATZ (43). Notably, MFI values for staining with 2C1 were reduced following treatments with either bafilomycin or MG132, with treated/untreated ratios below 1.0 (Fig. 5, *C* and *D*). There was no significant difference in the extent of reduction in CRT KO or CNX KO cells compared with WT cells (Fig. 5, *C* and *D*). The number of 2C1^+^ cells was also reduced (Fig. S5, C and D), whereas the same was not observed using eYFP fluorescence as the readout (Fig. S5, A and B). Thus, drug treatments appear to induce a greater loss in polymeric ATZ-expressing cells compared with untreated cells. The combined toxicities of drug treatment and polymeric ATZ accumulation could negatively influence the viability of drug-treated cells expressing polymeric eYFP-ATZ and also promote the accumulation of cells with low expression of polymeric eYFP-ATZ. To further verify the specificity of the 2C1 antibody for polymeric eYFP-ATZ, we compared 2C1 staining in Huh7.5 cells transfected with either eYFP-AAT or eYFP-ATZ at 48 h post-transfection. Whereas the averaged MFI values for 2C1 staining and % 2C1^+^ cells (of YFP^+^ cells) were higher for eYFP-ATZ transfected cells compared with the corresponding eYFP-AAT transfected cells, the most significant differences between eYFP-AAT and eYFP-ATZ transfections resulted from CNX KO condition (Fig. 5, *E* and *F* and Fig. S5, E and F). These findings suggest that, relative to the CRT KO, CNX KO causes more significant increases in cellular polymeric forms of eYFP-ATZ. However, the effect appears to be small, because there were no significant differences between CNX KO and WT cells in either the MFI values for 2C1 staining or % 2C1^+^ cells (of YFP^+^ cells) (Fig. 5*F* and Fig. S5F). Overall, these findings suggest that CNX deficiency results in enhanced polymeric ATZ accumulation but that the effect is small.

### CRT deficiency alters the ATZ interactome differently from CNX deficiency

To further understand the distinct functions of CRT and CNX chaperones in ATZ secretory trafficking, we analyzed the eYFP-ATZ interactomes in the Huh7.5 CRT and CNX KO cells relative to their WT counterparts. For these analyses, we used an anti-GFP single-chain nanobody (V_H_) coupled to agarose beads (GFP-Trap agarose, Chromotek) for immunoprecipitation (IP) of eYFP-ATZ, 24 h after transfection, and then performed MS to identify and compare interacting proteins.

**Figure 6:**
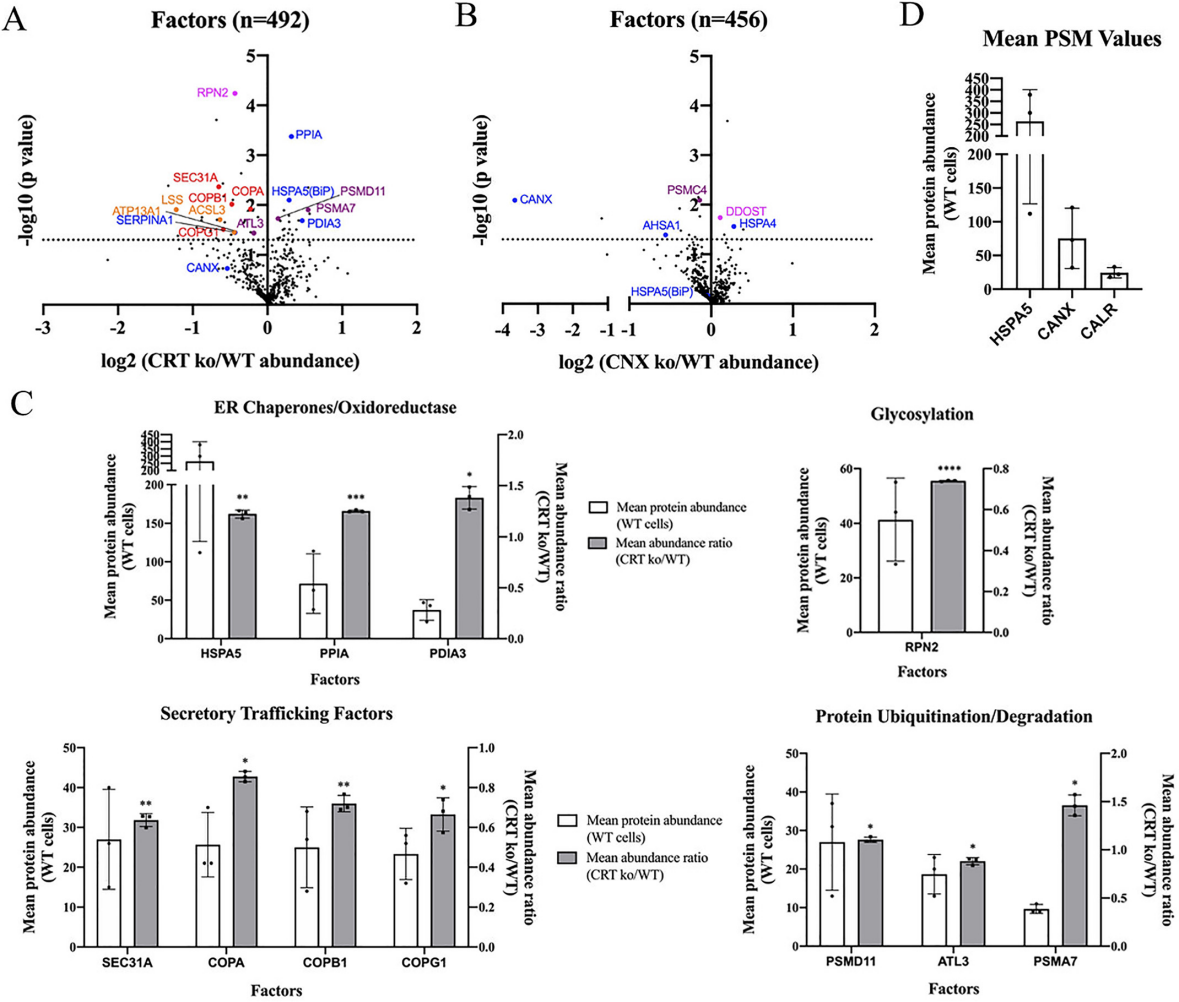
**MS reveals distinct alterations to the ATZ interactome induced by CRT deficiency compared with CNX deficiency.** eYFP-ATZ and associated factors were isolated from lysates of the indicated transfected Huh7.5 cells using GFP-Trap agarose beads and subjected to MS. *A*, volcano plot of all factors identified by MS, with PSM values ≥ 8 in the WT sample that were present in all three replicates of both CRT KO and WT Huh7.5 cells. The CRT KO/WT abundance ratio for each protein was calculated by dividing the specific PSM values of CRT KO cells by their corresponding PSM values from WT Huh7.5 cells and then normalizing relative to the total PSM ratio within each experiment: $\frac{\left(FactorNo.PSMsinCRTKO)/(FactorNo.PSMsinWT\right)}{\left(TotalNo.PSMsinCRTKO)/(TotalNo.PSMsinWT\right)}$. ER chaperones and oxidoreductases are shown in *blue*, factors involved in glycosylation are shown in *magenta*, factors involved in secretory trafficking are shown in *red*, factors involved in protein ubiquitination/degradation are shown in *purple*, and others are shown in *orange*. Data shown were obtained and averaged from three independent experiments. The *dotted line* indicates the significance threshold (*p* < 0.05). *p*-values were calculated based on log transformations of the normalized abundance ratios, followed by one-sample *t* tests of CRT KO and WT conditions. *B*, volcano plot of all factors as described and calculated in (*A*) identified in MS of eYFP-ATZ–transfected Huh7.5 CNX KO *versus* corresponding WT cells. Color coding of factors reflects that in *panel A*. Data were obtained and averaged from three independent experiments, and statistical analyses were performed as described in *panel A*. *C*, ER and related factors with abundance values significantly different between CRT KO and WT cells were sorted based on functions as ER chaperones/oxidoreductases, or those involved in glycosylation, secretory trafficking, or protein ubiquitination/degradation, and the mean protein abundance (mean PSMs) from WT cells and mean abundance ratios from three independent measurements (CRT KO/WT cells; the same calculations as the equation in *A*) are shown as *bar graphs* for each functional group. Indicated *p*-values were obtained as described in *panel A*. *p < 0.05, **p < 0.01, ***p < 0.001, ****p < 0.0001. *D*, protein abundance (mean PSM values) of recovered *HSPA5* (BiP), *CANX* (CNX), and *CALR* (CRT) from CRT WT cells are shown as mean ± S.D.

Peptide-spectrum match (PSM) values are a surrogate measure of protein abundance in MS analyses. We set ≥ 8 PSM in the WT sample as the cutoff for inclusion. We normalized the individual protein PSM values of the KO cells relative to WT cells (factor number of PSMs KO/factor number of PSMs WT) and further corrected for total PSM recovery differences between the two samples (total number of PSMs KO/total number of PSMs WT) per experiment. Based on this analysis, we identified 492 proteins that were present in three independent replicates of the MS analyses of CRT KO and corresponding WT cells (which were derived following transduction with a control virus at the same time the CRT KO cells were generated), of which there were 86 ER and related factors. Of these, 14 ER and related factors were significantly enhanced or reduced in their abundance ratios in CRT KO cells compared with WT cells (Fig. 6*A*). In the parallel analyses with CNX KO and corresponding WT cells (which were derived following transduction with a control vector at the time the CNX KO cells were generated), 456 factors were present in three independent replicates of the MS analyses in both CNX KO and WT cells. Among the ER and related factors, five were significantly enhanced or reduced in their abundance ratios in CNX KO cells compared with WT cells (Fig. 6*B*). Whereas CRT was detectable in WT cells (averaged PSM was 24.3) but undetectable in the CRT KO cells, CNX was present at an averaged abundance ratio of 0.08 in CNX KO cells, indicating that the CNX knockdown, although highly significant, was not complete. This accounts for CNX's presence in the volcano plot but with highly reduced abundance ratios in CNX KO cells (Fig. 6*B*) and the parallel absence of CRT in the corresponding CRT KO cells (Fig. 6*A*).

Importantly, CRT KO and CNX KO cells exhibited distinct changes in their eYFP-ATZ interactomes compared with corresponding WT cells (Fig. 6, *A* and *B*). Significant ER and related factors detected in CRT KO cells were sorted into functionally similar groups, including ER chaperones, oxidoreductases, factors involved in glycosylation, secretory trafficking, protein ubiquitination/degradation, or other. These groups are individually shown in bar graphs for clarity (Fig. 6*C*). Some ER chaperone–eYFP-ATZ interactions were enhanced in CRT KO cells. However, CNX and CLGN were down-regulated, although nonsignificantly in the case of CNX. CLGN is not shown in Fig. 6*A*, because it was not detected in 2/3 replicates of Huh7.5 CRT KO cells and was detected at lower abundance in the third replicate relative to the corresponding WT sample. On the other hand, CNX deficiency generally did not enhance the same chaperone–eYFP-ATZ interactions (Fig. 6*B*). Notably, eYFP-ATZ association with BiP (HSPA5), one of the most abundant eYFP-ATZ–interacting factors identified, was significantly enhanced in CRT KO cells (Fig. 6, *A* and *C*) but was decreased in CNX KO cells, although nonsignificantly (Fig. 6*B*). Additionally, eYFP-ATZ binding to PDIA3 (ERp57; an oxidoreductase and co-chaperone for both CRT and CNX) was significantly enhanced in CRT KO cells but was down-regulated in CNX KO cells, though nonsignificantly (not highlighted) (Fig. 6, *A* and *B*). On the other hand, interactions with HSPA4, a cytosolic chaperone, was enhanced in the absence of CNX (Fig. 6*B*). eYFP-ATZ interactions with factors relevant to ubiquitination (CAND1; nonsignificant) (44) and increased proteasome activity (PMSD11 and PMSA7) (45) were also enhanced in CRT KO cells relative to WT cells (Fig. 6*A*).

eYFP-ATZ associations with some factors related to ER-to-Golgi trafficking were significantly reduced in CRT KO cells (Fig. 6, *A* and *C*), including Sec31A, a component of the COPII complex, which mediates anterograde protein transport from the ER to the Golgi (46, 47). Furthermore, eYFP-ATZ interactions with α-COP, β-COP, and γ-COP, components of the COPI complex (48), which mediates retrograde transport from the Golgi to the ER, were also reduced in CRT-deficient cells. Thus, coincident with reduced eYFP-ATZ secretory trafficking, CRT deficiency significantly reduced eYFP-ATZ interactions with many factors relevant to ER-Golgi trafficking and ER export. In contrast, CNX KO cells did not show similar clusters of changes in eYFP-ATZ interactions with factors relevant to secretory trafficking (Fig. 6*B*). Notably, more pronounced changes in the interactions of eYFP-ATZ were observable in the absence of CRT, despite the relatively low abundance of CRT-eYFP-ATZ complexes compared with CNX-eYFP-ATZ complexes (Fig. 6*D*).

The analyses shown in Fig. 6 were done following correction for total PSM recovery differences (all factors) between the WT *versus* KO cells. Similar analyses were also undertaken after correction for total ATZ recovery differences, which were highly skewed toward factors with up-regulated CRT KO/WT ratios (Fig. S6A), likely because of the lower levels of recovery of ATZ in CRT KO cells compared with the WT cells (Table S2). This mode of normalization may thus over-correct for protein recovery differences. Despite this overall trend, decreased ATZ associations with the ER-to-Golgi anterograde trafficking component Sec31A was maintained, although nonsignificantly. Additionally, and notably, significantly lower levels of total Sec31A are measured in lysates of CRT KO cells compared with WT cells, by immunoblots (Fig. S7). However, we could not detect the interaction of Sec31A or other tested COPII components with eYFP-ATZ, indicating that the MS analysis is a more sensitive probe of ATZ interactions, including, as discussed below, those with CRT.

The overall trends of significantly increased associations of ATZ with various ER chaperones (Fig. 6*A*) were maintained following normalization for ATZ recovery (Fig. S6A). Additionally, several new factors linked to ubiquitination and proteasomal degradation were identified as up-regulated in CRT KO cells (Fig. S6). Despite these enhancements, CRT KO cells did not have an eYFP-ATZ degradative advantage (Fig. 5, Fig. S4, and Fig. S5).

### CRT deficiency alters the distribution of ATZ-ER chaperone complexes

**Figure 7:**
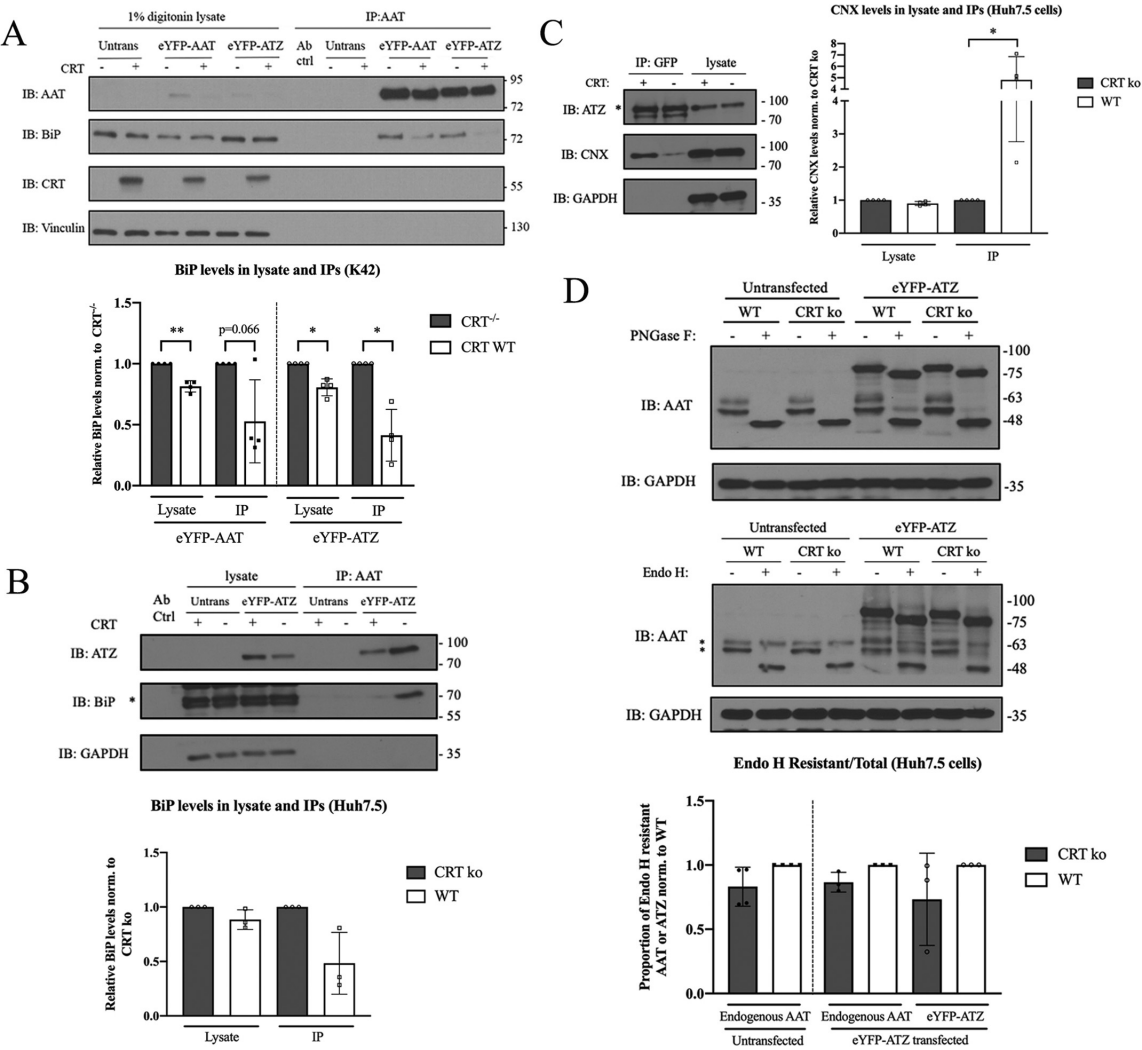
**CRT deficiency alters the distributions of ATZ complexes with ER chaperones.***A*, (*top*) BiP–eYFP-AAT or BiP–eYFP-ATZ interactions in K42 cells were visualized by immunoprecipitation following 1% digitonin lysis. Vinculin was used as a loading control. CRT interactions were not detectable in parallel IPs. *(Bottom)* densitometric quantification for total BiP and eYFP-AAT or eYFP-ATZ–co-immunoprecipitated BiP levels in CRT^−/−^ and CRT WT cells. Total BiP levels were calculated by dividing raw BiP intensities by their corresponding loading control intensities, whereas immunoprecipitated BiP levels were calculated by dividing immunoprecipitated BiP intensities by their corresponding immunoprecipitated ATZ intensities. The ratios were then normalized to CRT^−/−^ cells. Data were obtained from four independent transfections of one retroviral transduction and are shown as mean ± S.D. Paired one-sample *t* tests were performed on log-transformed data, comparing CRT^−/−^ and CRT WT conditions. *p < 0.05, **p < 0.01. *B*, (*top*) BiP–eYFP-ATZ interactions in Huh7.5 CRT KO (−) and WT (+) cells were visualized by indicated immunoprecipitation following 1% Triton lysis. GAPDH was used as a loading control. The *asterisk* indicates the BiP bands. (*Bottom*) densitometric quantifications for total BiP and eYFP-ATZ–co-immunoprecipitated BiP levels in CRT KO and WT cells as described in (*A*). Data were obtained from three independent transfections and are shown as mean ± S.D. Paired one-sample *t* tests were performed on log-transformed data comparing CRT KO and WT conditions. *C*, (*left*) CNX-eYFP-ATZ interactions in Huh7.5 CRT KO (−) and WT (+) cells were visualized by indicated immunoprecipitation following 1% Triton lysis. GAPDH was used as a loading control. The *asterisk* indicates the ATZ bands. *(Right)* densitometric quantifications for total CNX and eYFP-ATZ–co-immunoprecipitated CNX levels in CRT KO and WT cells as described in (*A*). Data were obtained from four independent transfections and are shown as mean ± S.D. Paired one-sample *t* tests were performed on log-transformed data comparing CRT KO and WT conditions. *p < 0.05. *D*, (*top and middle*) PNGase F and Endo H digestions in untransfected or eYFP-ATZ–transfected Huh7.5 cells. The two *asterisks* indicate two Endo H resistant bands of endogenous AAT in eYFP-ATZ–transfected cells. *(Lower)* densitometric quantification for Endo H resistant endogenous AAT of the total AAT levels in untransfected or eYFP-ATZ–transfected cells, or Endo H resistant eYFP-ATZ levels of the total ATZ levels in CRT KO and WT cells. The ratios were normalized to WT cells. Data were obtained from three independent experiments and are shown as mean ± S.D. Paired one-sample *t* tests were performed on log-transformed data comparing CRT KO and WT conditions.

Normalization for either total protein recovery (Fig. 6*A*) or ATZ recovery (Fig. S6A) suggested that CRT deficiency induced ATZ binding to some ER chaperones. We further validated these results using co-immunoprecipitation analyses. HSPA5 (BiP) was one of the most abundant eYFP-ATZ–interacting factors identified by the MS analyses, and CRT KO cells displayed enhanced BiP recovery relative to WT cells (Fig. 6*A*). In K42 cells (Fig. 7*A*), whereas there was a ∼20% reduction in BiP lysate levels in CRT WT expressing cells relative to CRT^−/−^ cells, consistent with previous findings (49), this reduction was significantly smaller than the reduction in eYFP-ATZ–co-immunoprecipitated BiP in the presence of CRT (there was an almost a 2-fold decrease in the amount of co-immunoprecipitated BiP in cells expressing CRT WT compared with CRT^−/−^ K42 cells) (Fig. 7*A*, *lower panel*). eYFP-ATZ–co-immunoprecipitated BiP levels were also higher in Huh7.5 CRT KO cells (Fig. 7*B*). Thus, consistent with the MS results (Fig. 6*A*), these results verify increased BiP–eYFP-ATZ interaction under CRT KO conditions. Consistent with the MS data, the eYFP-ATZ–co-immunoprecipitated CNX level was also reduced in CRT KO cells compared with WT, although the total CNX level in the lysate was up-regulated (Fig. 7*C*). In parallel analyses, we were unable to detect CRT–eYFP-ATZ interactions in co-immunoprecipitations in either CRT KO or WT cells, consistent with the MS analyses which yielded higher PSM values for both BiP and CNX compared with CRT (averaged PSM values for BiP, CNX, and CRT were 263.6, 75.3, and 24.3, respectively) (Fig. 6*D*).

Because RPN2, a component of the oligosaccharyl transferase complex, was found to be significantly reduced in the eYFP-ATZ interactomes of CRT KO relative to WT Huh7.5 cells (Fig. 6*A*), we also compared the glycosylation state and glycan compositions of eYFP-ATZ from CRT KO and WT cells. Lysates from untransfected and eYFP-ATZ–transfected Huh7.5 cells were treated with PNGase F to assess overall glycosylation levels. The majority of detectable eYFP-ATZ was glycosylated in both CRT KO and WT cells, indicating that glycosylation is largely unaltered (Fig. 7*D*, *top panel*). eYFP-ATZ in lysates was also compared for sensitivity to Endoglycosidase H (Endo H), an enzyme that cleaves high-mannose glycans between their two GlcNAc residues within the core of the glycan chain (50). *N*-glycosylated proteins become resistant to Endo H when they traffic through the Golgi and undergo glycan modifications of their terminal mannose residues. We found that both endogenous AAT from untransfected cells and eYFP-ATZ in transfected cells show reduced Endo H–resistant fractions in CRT KO cells (Fig. 7*D*, *middle and bottom panels*) but that the differences were not significant, at least in the steady state. Notably however, in cells expressing eYFP-ATZ, the glycosylation pattern of endogenous AAT is altered, with two Endo H–resistant species compared with a single higher molecular weight Endo H–resistant band in the absence of eYFP-ATZ expression (Fig. 7*D*, *middle panel*, the two bands indicated by *asterisks*). The lower band is over-represented in the presence of CRT and may correspond to under-glycosylated species that arise because of either incomplete glycosylation of *N*-linked glycan sites or incomplete glycan modifications in the Golgi. Enhanced binding to CNX or CRT may sterically block one or both modifications.

Together, the findings of Figure 6, Figure 7 indicate that BiP is a major eYFP-ATZ–interacting factor. BiP–eYFP-ATZ interactions are elevated in CRT KO cells as identified both by MS and by co-immunoprecipitation/immunoblotting, coincident with reduced secretory trafficking of eYFP-ATZ. Additionally, eYFP-ATZ binding to both CNX and CLGN is reduced in Huh7.5 CRT KO cells, suggesting that both of these chaperone-substrate interactions are compromised in CRT-deficient cells, consistent with previous literature showing that CNX function is reduced when CRT is absent (49).

### CRT deficiency enhances cellular ATZ inclusion body formation

**Figure 8:**
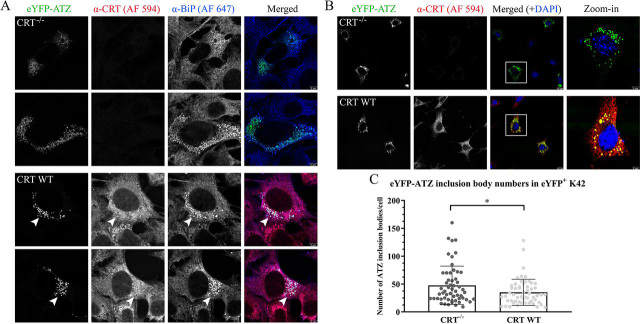
**CRT deficiency enhances formation of ATZ IBs, which contain both CRT and BiP.***A*, representative immunofluorescence images demonstrating CRT-BiP–eYFP-ATZ colocalization within eYFP-ATZ inclusion bodies (IBs) (shown as *white arrows*) at 48 h post-transfection in K42 cells. The cells were fixed and stained with antibodies against CRT and BiP with eYFP fluorescence as the indicator for ATZ. 4′,6-diamidino-2-phenylindole was used as a nuclear stain. Scale for merged images is 5 µm. *B*, representative immunofluorescence images that show eYFP-ATZ IBs colocalized with CRT, when present, in eYFP-ATZ–transfected cells, at 48 h post-transfection in K42 cells. 4′,6-diamidino-2-phenylindole was used as a nuclear stain. Scale for merged images is 10 µm and *inset* is 5 µm. *C*, quantification of the number of ATZ inclusion bodies shown in (*B*). A total of 56 cells were quantified over five independent transfections, and data are shown as mean ± S.D. Unpaired two-tailed *t* test was performed. *p < 0.05. *DAPI*, 4′,6-diamidino-2-phenylindole.

**Figure 9:**
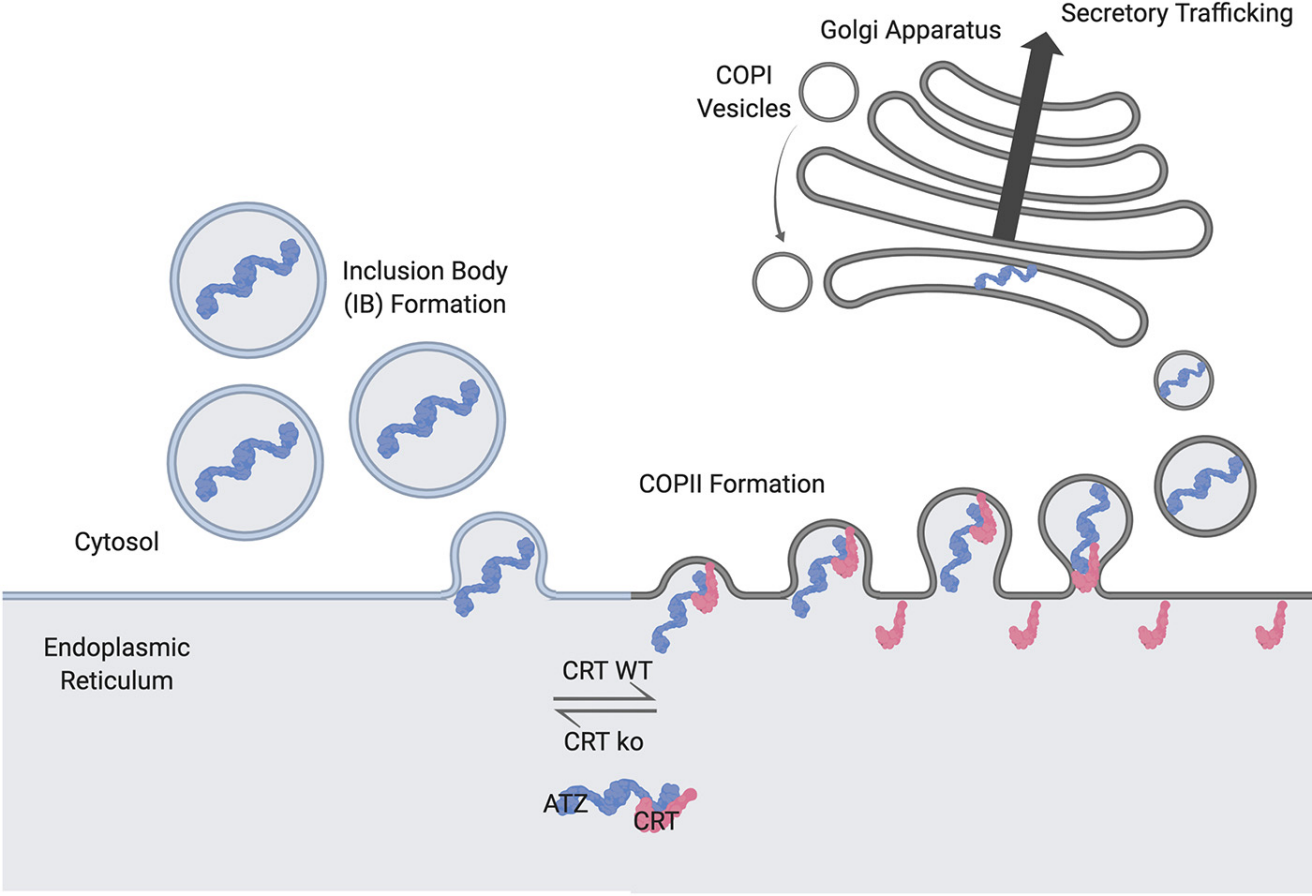
**Calreticulin increases secretory trafficking of ATZ, whereas CRT deficiency enhances IB formation.** CRT increases the secretory trafficking of ATZ via two proposed mechanisms. These include (*A*) direct interactions with ATZ to promote ATZ folding and reduce insolubility and (*B*) facilitation of ATZ interactions with COPII components, either by reducing associations with ER chaperones that inhibit incorporation into COPII vesicles or by increasing Sec31A levels and the resulting assembly of large COPII vesicles needed for the secretory trafficking of polymeric ATZ. Impaired secretory trafficking in CRT-deficient cells enhances the generation of fragmented ER-derived vesicles (IBs) that contain ATZ and BiP, to maintain the cell's secretory homeostasis. The illustration was generated using BioRender.

Inclusion body (IB) formation is a histopathological hallmark of ATD in liver cells (16), suggested to be a cell-protective mechanism to sequester misfolded proteins such that glycoprotein processing and secretion can still occur (31). IBs are vesiculated, electron-dense, ER-derived structures that arise in parallel with loss of the ER branching tubule network (51). They are separated from the main ER network and thought to represent fragmented ER (37). Our findings thus far indicate that CRT deficiency impairs the secretory trafficking of eYFP-ATZ, coincident with enhanced binding to ER chaperones and reduced eYFP-ATZ binding to cellular components important for ER-Golgi trafficking. We further examined whether the impairment in secretory trafficking in CRT-deficient cells also coincides with ATZ IB formation. Previous studies have indicated that several ER proteins are present within ATZ IBs (31, 37). Indeed, in colocalization analyses, we found that both BiP and CRT colocalize with eYFP-ATZ within structures resembling IBs in K42 cells at 48 h post-transfection (Fig. 8*A*). We further quantified the effects of CRT expression on cellular IB formation using confocal microscopy. We found that CRT expression significantly diminished the number of eYFP-ATZ–containing IBs in K42 cells (Fig. 8, *B* and *C*), which were chosen for microscopy because they form a uniform monolayer consisting of uninucleate cells. These findings indicate a reciprocal relationship between secretory trafficking of eYFP-ATZ and IB formation. The presence of CRT promotes the former and inhibits the latter (Fig. 9).

## Discussion

As a secreted glycoprotein, ATZ enters the ER via the translocon pore where it undergoes glycosylation and glucose trimming by glucosidase I and II, permitting interactions with the lectin chaperones CRT and CNX. Whereas roles for CNX in the folding and degradation of AAT and its variants are well-studied and reported in the literature (22, 23, 24, 25, 26, 27, 28, 29, 30, 31, 32, 33), interactions with CRT are poorly studied. In Huh7.5 cells, CRT's interactions with eYFP-ATZ are detectable by MS analyses and result in a shift in the fraction of the protein that gets secreted, a function that is specific to CRT (Figure 1, Figure 2, Figure 3, Figure 4, Figure 5, Figure 6).

A recent study has reported that CRT knockdown results in enhanced ATZ secretion in an ERdj3-dependent manner (52), findings that are contrary to our studies described here. It is important to note that our experiments use a fluorescence-based ratiometric method wherein we quantify the fraction of the total protein pool that is secreted. In our MS analyses, although ERdj3 is detected in the eYFP-ATZ interactome (data not shown), it is not an ER factor identified as displaying significantly altered abundance in the eYFP-ATZ interactome of CRT KO cells compared with WT cells. Additionally, the use of embryonic deletions and CRISPR/Cas9-based knockouts rather than siRNA knockdowns used by Khodayari *et al.* (52) enabled us to eliminate any effects arising from the fraction of CRT still being expressed in the cells.

CRT interacts with glycoproteins using several conserved residues in its glycan-binding site, of which Tyr-92 is crucial (35, 36). We show that the Y92A mutant is less effective at inducing an elevated media:cell fluorescence ratio of eYFP-ATZ relative to CRT WT (Fig. 1). These findings indicate that CRT-mediated enhancement of secretion is partly dependent on its glycan-binding site and therefore its conventional chaperone activity. The findings are also consistent with a role for CRT in mediating the early glycan-dependent folding of ATZ, failure of which, exhibited in the case of CRT KO cells, would increase interactions with other ER chaperones, including, rather prominently, BiP, and reduce ATZ selection for export from the ER. Consistent with these findings, previous studies have shown that the presence of UDP-glucose glycoprotein glucosyltransferase (UGGT1; the enzyme responsible for monoglucosylation of AAT/ATZ) and CRT enhances recovery of soluble forms of another AAT variant, the NHK mutant.

The conventional chaperone activity of CRT does not fully explain its functions in eYFP-ATZ secretory trafficking, because the Y92A mutant only partially abrogated the positive influences of CRT on secretory trafficking. Furthermore, CRT–eYFP-ATZ complexes display reduced abundance in the steady state relative to CNX–eYFP-ATZ complexes (Fig. 6*D*), yet it is CRT rather than CNX that has a strong positive effect on eYFP-ATZ secretory trafficking. eYFP-ATZ associations with factors involved in secretory trafficking are reduced in CRT KO cells (Fig. 6, *A* and *C*), consistent with the parallel reduction in the media:cell fluorescence ratio of eYFP-ATZ (Figure 1, Figure 2, Figure 3). Whereas the LMAN1-MCFD2 cargo receptor complex is known to be important for AAT secretion (53), standard COPII vesicles, with an average diameter of just 60–90 nm, may be too small to incorporate eYFP-ATZ polymers. Additional cellular factors are likely required for creation of larger COPII vesicles, the generation of which may be facilitated by CRT. Previous studies indicate that the calcium-dependent ubiquitination of Sec31A is required for the secretion of specific types of collagens (54), which also require large-diameter COPII vesicles for their secretion (47). Because CRT contains low-affinity calcium-binding sites in its C-terminal domain, which contributes to ER and cellular calcium homeostasis and calcium release from the ER (8, 9, 10), it is possible that altered ER calcium levels in CRT-deficient cells affect eYFP-ATZ secretory trafficking via Sec31A. We find that total Sec31A levels are significantly reduced in CRT KO cells (Fig. S7), which supports the above hypothesis, despite the inability to detect interactions of COPII components with eYFP-ATZ by co-immunoprecipitation/immunoblotting (Fig. S7). The Y92A mutant of CRT retains the low-affinity calcium-binding sites found at the C terminus of CRT. The ability of CRT Y92A to partially restore eYFP-ATZ secretory trafficking (Fig. 1) might be attributable to its intact calcium-binding functions. Alternatively, it is possible that CRT directly or indirectly enhances ATZ incorporation into COPII vesicles via other mechanisms. For example, enhanced ER chaperone associations in CRT-deficient cells could inhibit binding sites important for interaction with specific cargo receptors. These mechanisms require further investigation.

Reduced secretory trafficking of eYFP-ATZ, exhibited in CRT KO cells, results in greater incorporation of eYFP-ATZ into IBs (Fig. 8). Although there are few mechanistic insights into the biogenesis of IBs, these data indicate a reciprocal relationship between successful secretory trafficking and IB incorporation of ATZ (Fig. 9). The inability to successfully incorporate into COPII vesicles could ultimately result in fragmented ER vesicles (IBs) that are physically dislodged from the tubular ER network to maintain the cell's secretory homeostasis (31, 37). The prolonged maintenance of ATZ within IBs that also contain BiP (Fig. 8*A*) could also lead to the observed induction of BiP–eYFP-ATZ binding in the steady state in CRT-deficient cells (Fig. 6*A* and Fig. 7, *A* and *B*). CRT deficiency results in less-efficient eYFP-ATZ binding to CNX and CLGN (Fig. 6*A* and Fig. 7*C*), both of which belong to the CNX structural family of chaperones (6). However, CRT but not CNX sufficiency is important for more productive eYFP-ATZ trafficking through the secretory pathway (Fig. 1, Fig. 3, and Fig. 4).

Notably, despite structural similarities between CRT and CNX, the two chaperones play distinct roles in ATZ folding, degradation, and secretory trafficking. In contrast to CRT deficiency (Figure 1, Figure 2, Figure 3, Figure 4, Figure 5, Figure 6), CNX deficiency does not enhance the secretory trafficking of eYFP-ATZ (Fig. 4) or increase BiP–eYFP-ATZ interaction (Fig. 6). A functional consequence of CNX deficiency is the enhanced accumulation of polymeric forms of eYFP-ATZ, as measured with the 2C1 antibody (Fig. 5). Using inhibitors of the proteasome or the lysosomal acidification machinery, we ascertained that CRT does not have a major role in eYFP-ATZ proteasomal or lysosomal degradation (Fig. 5, Fig. S4, and Fig. S5), consistent with previous findings (22). On the other hand, a key role for CNX in the ERLAD pathway has been suggested, via the ATZ-CNX-FAM134B complex (22). Based on these studies, it is expected that CNX-deficient cells will accumulate polymeric ATZ at enhanced levels, consistent with the analyses of Fig. 5, *E* and *F*. The extreme sensitivity of 2C1^+^ cells to either bafilomycin or MG132 treatments, indicated by the reduced number of 2C1^+^ cells (Fig. S5) and reduced MFI of 2C1 staining following drug treatments (Fig. 5), suggests that excessive accumulation of polymeric eYFP-ATZ is toxic to cells. Furthermore, the ability to recover 2C1^+^ cells under CNX-deficient conditions (Fig. 5) suggests that there are multiple redundant pathways for polymeric eYFP-ATZ disposal or sequestration. Our MS data also indicates a reduction in the extent of BiP–eYFP-ATZ binding under conditions of CNX deficiency (Fig. 6*B*). Because eYFP-ATZ and BiP are localized within IBs (Fig. 7), further studies are needed to examine how CNX deficiency affects inclusion body formation and the overall subcellular distributions of ATZ.

Overall, through this study, we show that CRT's role in eYFP-ATZ clearance may be multiple-fold (Fig. 9). In addition to direct Tyr-92–dependent interactions with eYFP-ATZ (Fig. 1), CRT enhances eYFP-ATZ associations with other lectin chaperones (CNX and CLGN) (Figure 6, Figure 7), which could collectively enhance folding and reduce insolubility, and increases eYFP-ATZ trafficking out of the ER (Figure 1, Figure 2, Figure 3), ultimately resulting in more eYFP-ATZ being secreted and reduced IB incorporation (Figure 8, Figure 9). A second important component of CRT's effects on ATZ secretory trafficking may be the direct or indirect facilitation of enhanced eYFP-ATZ interaction with large COPII vesicles required for eYFP-ATZ secretion, through effects on Sec31A and cellular calcium homeostasis (Fig. 9). The relatively low abundance of CRT in the eYFP-ATZ interactome supports the model of both direct and indirect roles for CRT in eYFP-ATZ secretory trafficking. In contrast to CRT's role, CNX, likely via a role in ERLAD (22), leads to polymeric eYFP-ATZ clearance, and its deficiency results in increased accumulation of polymeric eYFP-ATZ (Fig. 5). In sum, our efforts contribute to the broader knowledge of ER chaperone–mediated effects on ATZ trafficking, with a focus on CRT-specific effects. Further understanding of the factors that control the secretory trafficking of ATZ is important toward the clinical treatment of ATD, with discoveries benefitting those suffering from lung and liver malfunction due to faulty trafficking of defective AAT.

## Experimental procedures

### Antibodies and reagents

All reagents were obtained from Thermo Fisher Scientific unless otherwise stated. Other reagents used were MG132 (Sigma-Aldrich, 474787), bafilomycin A1 (Cayman Chemical, 11038), PNGase F and Endo H (New England Biolabs, P0704 and P0702), paraformaldehyde (PFA) (Electron Microscopy Services, 15714), Enzyme-Free Cell Dissociation Buffer and TrypLE (Gibco, 13151014 and 12605010), and Protein G Sepharose beads (Abcam, ab193259). Primary antibodies used were anti-AAT (rabbit polyclonal IgG, Agilent Dako, A0012), anti-human AAT (polymer-selective) (2C1; mouse monoclonal IgG1, Hycult Biotech, HM2289), anti-CRT (rabbit polyclonal IgG, Thermo Fisher Scientific, PA3-900), anti-CNX (rabbit polyclonal IgG, Enzo Life Sciences, ADI-SPA-860), anti-CNX (C5C9; rabbit monoclonal, Cell Signaling Technology, 2679), anti-GAPDH (14C10; rabbit monoclonal IgG, Cell Signaling Technology, 2118), anti-GFP (GF28R; mouse monoclonal IgG1, Thermo Fisher Scientific, MA5-15256), anti-GRP78/BiP (rabbit polyclonal IgG, Abcam, ab21685), anti-ubiquitin (P4D1; mouse monoclonal IgG, Cell Signaling Technology, 3936), anti-Vinculin (E1E9V; rabbit monoclonal IgG, Cell Signaling Technology, 13901), anti-Sec31A (mouse monoclonal IgG1, BD Biosciences, 612350), anti-Sec24A (rabbit, Cell Signaling Technology, 9678), and anti-Sec24C (D9M4N, rabbit monoclonal IgG, Cell Signaling Technology, 14676). For GFP immunoprecipitation, we used GFP-Trap agarose (alpaca single-chain V_H_H nanobody coupled to agarose beads, Chromotek, gta-20). For immunostaining applications, anti-mouse BiP antibody was custom-made at GeneTel Laboratories from chickens immunized with the CGEEDTSEKDEL peptide and affinity purified. Secondary antibodies used for immunoblotting were HRP-conjugated goat anti-rabbit IgG (H + L), mouse anti-rabbit IgG (light chain–specific), or goat anti-mouse IgG (H + L) (all from Jackson ImmunoResearch, 111-035-144, 211-032-171, and 115-036-062). For immunostaining and/or flow cytometry, secondary antibodies used were Alexa Fluor 405-conjugated goat anti-mouse IgG (H + L) (Abcam, ab175661), Alexa Fluor 555-conjugated goat anti-mouse IgG1 (Thermo Fisher Scientific, A-21127), Alexa Fluor 594-conjugated goat anti-rabbit IgG (H + L) (Cell Signaling Technology, 8889), Alexa Fluor 647-conjugated goat anti-chicken IgY (Abcam, ab150175), and APC/Cyanine7 goat anti-mouse IgG (Poly4053; BioLegend, 405316). All secondary antibodies chosen had minimal cross-reactivities with other species.

### DNA constructs

eYFP-AAT and eYFP-ATZ, previously characterized (37), were a kind gift from Dr. Stefan Marciniak, University of Cambridge. Starting at the second amino acid position of the mature protein, AAT in eYFP-AAT construct had an identical protein sequence to several AAT sequences in the NCBI database, including NP_000286.3. On the same background, the eYFP-ATZ sequence has the E342K mutation.

Human CRT (GenBank ID BC020493.1) was cloned into pMSCV puro vector (Clontech Laboratories) between the BglII and XhoI sites. The Y92A mutant of hCRT was generated using Quikchange II XL site-directed mutagenesis (Agilent) with the primers 5′-GGAAACAGCTTCACAGCGCCGCCCCCACAGTC-3′ and 5′-GACTGTGGGGGCGGCGCTGTGAAGCTGTTTCC-3′ per the manufacturer's instructions. For generation of CRISPR/Cas9 knockouts, pLentiCRISPR v2 blasticidin (Addgene) vector was used. Guide RNAs were designed using Broad Institute's GPP Web Portal against human *Calr* gene (5′-CATGAGCAGAACATCGACTG-3′ against exon 3 and 5′-GGCCACAGATGTCGGGACCT-3′ against exon 6) and human *Canx* gene (5′-CACCGTTTAGAAAGCAGTTTCACAT-3′ against exon 6) and cloned into the vector as per depositor's protocol. Sanger sequencing was performed at the University of Michigan DNA Sequencing Core to verify the sequences of all constructs used.

### Mammalian cell culture and transient transfections

*Calr*^−/−^ mouse embryonic fibroblasts (MEFs) (K42 cells) (gifted by Dr. Marek Michalak, University of Alberta, Canada, first described in (9)) were maintained in RPMI 1640 medium supplemented with 10% FBS and 1× antibiotic-antimycotic. Huh7.5.2 (referred to as Huh7.5 throughout) human hepatocellular carcinoma-derived cells (gifted by Dr. Andrew Tai, University of Michigan) and HEK 293T cells were maintained in DMEM supplemented with 10% FBS and 1× antibiotic-antimycotic and passaged as required. All cell lines were grown at 37 °C and 5% CO_2_. For transient transfections in K42 and Huh7.5 cells, 2.5–3 × 10^5^ cells were seeded 1 day prior to transfection on a 6-well plate and spent media was replaced with fresh media 30 min to 1 h prior to transfection. Lipofectamine 3000 (Thermo Fisher Scientific) and DNA were diluted in Opti-MEM as per the manufacturer's instructions and the resulting transfection mix was added to the cells. Media was replaced 6–8 h post-transfection.

### Flow cytometry

The cells were collected using Enzyme-Free Cell Dissociation Buffer (Gibco) or TrypLE (Gibco) and washed once in prewarmed PBS. The cells were then stained with Aqua Live/Dead stain (Thermo Fisher Scientific) for 10 min at room temperature (RT) and washed with PBS. This was followed by fixing with 4% PFA in PBS and washing with PBS. In some cases of eYFP fluorescence estimation, the samples were analyzed after this step. In other experiments involving 2C1 antibody staining for polymeric ATZ, the cells were permeabilized using 0.2% saponin in FACS buffer (PBS + 2% FBS), followed by incubation in the indicated primary antibody (used at a dilution of 1:100) in 0.2% saponin in FACS buffer for 30 min at 4 °C. The cells were then washed with FACS buffer and incubated with secondary antibody (used at a dilution of 1:200) in 0.2% saponin in FACS buffer for 15 min at RT. As a control, transfected cells were also stained with secondary antibody alone in the absence of primary antibody. Following incubation, the cells were washed twice with FACS buffer and analyzed. For experiments with proteasomal and lysosomal inhibitors, 4–6 × 10^5^ cells were seeded on a 6-well plate. The next day, the cells were transfected with eYFP-ATZ–encoding plasmid using Lipofectamine 3000 (Thermo Fisher Scientific) as described. At 20 h post-transfection, media was changed and either 100 nm bafilomycin A1 or 10 μg/ml MG132 was added. At 24 h post-transfection, the cells were harvested using the flow cytometry protocol mentioned above.

Flow cytometry files were analyzed using FlowJo v10. For eYFP quantifications, the cells were gated on forward and side scatter, live cells, then on YFP^+^ cells, based on the untransfected cell signal as a negative control. Cell counts, eYFP MFI, and % eYFP^+^ (of live cells) were then obtained based on the gate. For 2C1 quantifications, the cells were first gated on forward and side scatter, live cells, and YFP^+^ cells. The negative boundary for 2C1 staining was then set based on the secondary antibody alone control to gate on 2C1^+^ cells for further quantifications of 2C1 MFI and % 2C1^+^ cells.

### Estimation of media and cellular eYFP fluorescence

48 h post-transfection, media was collected and centrifuged at 16,250 × *g* for 10 min at 4 °C to remove cell debris. Media fluorescence was estimated using FluoroMax 4 (Horiba) spectrofluorimeter with the excitation filter set at 514 nm and emission filter set at 527 nm. Media fluorescence of untransfected cells was used for background subtraction for both eYFP-AAT and eYFP-ATZ transfections. The cells were collected, washed once in 1× PBS, and used for flow cytometry. The samples were processed on a Fortessa (BD Biosciences) flow cytometer using the FITC channel to measure eYFP fluorescence. eYFP MFI and cell counts were obtained using FlowJo v10. Media:cell fluorescence ratio was calculated as $\frac{Mediafluorescence(backgroundsubtracted)}{CellulareYFPMFI\times Numberof{eYFP}^{+}cells}$.

### Retroviral infection for CRT expression in K42 cells

HEK 293T cells were transfected with the appropriate transfer, packaging, and envelope plasmids using X-tremeGENE (Sigma-Aldrich) or Lipofectamine LTX (Thermo Fisher Scientific) for retroviral infections as outlined in (36). 72 h post-transfection, supernatants were collected, filtered using a 0.45-μm filter, and added to cells. Media was changed the following day and selection was started 72 h post-transduction with 2 μg/ml puromycin. Transductions were verified by immunoblotting.

### Gene knockouts

Protocols were modified from (55). To produce lentivirus, in a T-75 flask, HEK 293T cells at ∼80% confluency were transfected with packaging and transfer plasmids using Lipofectamine LTX and Plus reagents (Thermo Fisher Scientific) in Opti-MEM (Gibco). For both CRT and CNX KO, control plasmids lacking guide RNA were used in a parallel set of transfections to generate WT controls for each cell line. After 60 h of growth in DMEM supplemented with 10% FBS, virus-containing media was collected, spun down to pellet any debris, and filtered through a 0.45-μm low protein binding membrane. For some transductions, the viral media was further concentrated using a 15-ml 30K MWCO Amicon Ultra centrifugal filter unit (Sigma-Aldrich). Huh7.5 cells were seeded to ∼80% confluency on a 6-well plate. Viral media with 8 μg/ml polybrene was added to cells, and transduction was achieved by spinoculation in plates via centrifugation at 1,000 × *g* for 2 h at 37 °C. Approximately 4 h after the addition of virus and polybrene, 80% of the media was changed (DMEM supplemented with 10% FBS and 1× antibiotic-antimycotic used thereafter). 72 h later, media was changed and selection was started using 8 μg/ml blasticidin. Following selection, knockdown was visualized following cell lysis, SDS-PAGE, and immunoblotting with anti-CRT (Thermo Fisher Scientific, PA3-900) or anti-CNX (Cell Signaling Technology, 2679) antibody. In the case of CRT knockouts, lentiviral infections were repeated thrice to ensure complete knockout. Limiting dilutions did not work at low seeding densities in Huh7.5 cells.

### Cell lysis

For all experiments involving cell lysis, except where indicated, 1% Triton X-100 in 1× TBS (50 mm Tris-Cl, 150 mm NaCl, and 1 μm CaCl_2_, pH 7.4) with Complete Mini (Roche) or Protease Inhibitor mixture 1:100 (Sigma-Aldrich, 8340) was used. The cells were washed in ice-cold PBS and lysed in the lysis buffer for 30 min at 4 °C with gentle agitation or on ice. Lysates were then cleared of debris by high-speed centrifugation (16,250 × *g*) for 30 min at 4 °C. BCA assays (Thermo Fisher Scientific) were performed to normalize protein loads for further downstream experiments.

### Immunoprecipitation (IP) and co-immunoprecipitation

The cells were collected using Enzyme-Free Cell Dissociation Buffer (Gibco) or TrypLE (Gibco). The cells were washed in ice-cold PBS and cross-linked in 1 ml of 2 mm Dimethyl dithiobispropionimidate (DTBP) peroxide (in PBS) for 50 min on ice, followed by incubation in 50 μl of 1 m Tris-HCl (pH 7.5) for 10 min on ice to quench the cross-linking reaction. For BiP-AAT co-immunoprecipitations in K42 cells, the cells were lysed in 1% digitonin in 50 mm HEPES, 150 mm NaCl, and 1 μm CaCl_2_ (pH 7.4) with Complete Mini (Roche) for 20 min on ice. For BiP-AAT and anti-GFP co-immunoprecipitations in Huh7.5 cells, the cells were lysed in 1% Triton X-100 lysis buffer as described above. Lysates were cleared by centrifugation as described, and BCA-normalized amounts of protein were used for immunoprecipitation. 1 mg of cell lysate in 1 ml of lysis buffer was incubated with 7.3 μg of anti-AAT polyclonal antibody (Agilent Dako, A0012) overnight at 4 °C with gentle agitation. After incubation, Protein G Sepharose beads (Abcam) were added for 1 h at 4 °C with gentle agitation. Beads were then washed thrice with 0.1% Triton X-100 in cold 1× TBS and boiled in 5× Laemmli buffer to elute immunoprecipitated protein. For anti-GFP co-immunoprecipitations in Huh7.5 cells, cell lysate (typically 1 ml of 1 mg/ml total protein) was incubated with 15 μl of GFP-Trap agarose resin (Chromotek, gta-20) for 1–1.5 h at 4 °C with gentle agitation. Beads were then washed once with cold 0.1% Triton X-100 in TBS and twice with cold 1× PBS. Beads were boiled in 5× Laemmli buffer for immunoblotting, or were stored at −80 °C for MS submission.

### MS

The GFP-Trap agarose beads from the IP procedure described above (of 4 mg of total cell lysate) were resuspended in 50 μl of 0.1 m ammonium bicarbonate buffer (pH 8). 50 μl of 10 mm DTT were added and samples were incubated at 45 °C for 30 min to reduce cysteines. The samples were incubated with 65 mm 2-chloroacetamide in the dark for 30 min at RT for alkylation of cysteines. The samples were then digested overnight with 1 μg of sequencing grade, modified trypsin at 37 °C with constant shaking in a Thermomixer. Digestion was stopped by acidification and peptides were desalted using SepPak C18 cartridges using the manufacturer's protocol (Waters Corp.). The samples were completely dried using vacufuge. Resulting peptides were dissolved in 8 μl of 0.1% formic acid/2% acetonitrile solution. 2 μl of the peptide solution were resolved on a nano-capillary reverse phase column (Acclaim PepMap C18, 2 micron, 50 cm, Thermo Fisher Scientific) using a 0.1% formic acid/2% acetonitrile (buffer A) and 0.1% formic acid/95% acetonitrile (buffer B) gradient at 300 nl/min over 180 min (2–22% buffer B for 110 min, 22–40% buffer B for 25 min, and 40–90% buffer B for 5 min, followed by holding in 90% buffer B for 5 min and reequilibration with buffer A for 25 min). Eluent was directly introduced into the Orbitrap Fusion tribrid mass spectrometer (Thermo Fisher Scientific) or into a Q Exactive HF mass spectrometer (Thermo Fisher Scientific) using an EasySpray source. MS1 scans were acquired at 120,000 resolution (automatic gain control (AGC) target = 1 × 10^6^; max injection time (IT) = 50 ms) or at 60,000 resolution (AGC target = 3 × 10^6^; max IT = 50 ms). Data-dependent collision-induced dissociation MS/MS spectra were acquired using the Top speed method (3 s) following each MS1 scan (normalized collision energy (NCE) ∼28–32%; AGC target = 1 × 10^5^; max IT = 45 ms). Proteins were identified by searching the MS/MS data against *Homo sapiens* (UniProt; 20,353 reviewed entries; downloaded on 06-29-2019) using Proteome Discoverer (v2.4, Thermo Fisher Scientific). Search parameters included MS1 mass tolerance of 10 ppm and fragment tolerance of 0.2 Da; two missed cleavages were allowed; carbamidomethylation of cysteine was considered as fixed modification and oxidation of methionine; deamidation of asparagine and glutamine were considered as potential modifications. False discovery rate was determined using Percolator and proteins/peptides with a false discovery rate of ≤1% were retained for further analysis.

Three independent technical MS replicates were performed of samples from GFP-Trap IPs from lysates of eYFP-ATZ–transfected CRT KO and the corresponding WT Huh7.5 cells. Three independent technical MS replicates were separately performed of GFP-Trap IPs from lysates of eYFP-ATZ–transfected CNX KO and the corresponding WT Huh7.5 cells. Replicates of 3 were used to calculate statistically significant differences in ATZ associations. PSM values are indicative of protein abundance. A cutoff of ≥ 8 PSM in the WT condition was used to determine factors to be considered for further analyses to focus on the most abundant factors, and only factors that were present in all three replicates, for each cell type (CRT or CNX KO and WTs), were further considered. The KO/WT abundance ratio for each factor was calculated by dividing the specific PSM values from KO cells by their corresponding PSM values from WT cells and then correcting for total protein PSM differences between the WT cells and KO cells using the following calculation:

$$\frac{\left(FactorNo.ofPSMsinCRTKO)/(FactorNo.ofPSMsinWT\right)}{\left(TotalNo.ofPSMsinCRTKO)/(TotalNo.ofPSMsinWT\right)}$$

The corrected abundance ratios were compiled from the three independent replicates and were log-transformed. Statistical analyses of factors were then performed using paired two-tailed *t* tests in Microsoft Excel to identify statistically significant factors. *p*-values and means were log-transformed (base 10 and base 2, respectively) for volcano plots. *p*-values ≤ 0.05 were considered statistically significant. The list of significant factors was further manually sorted for ER-relatedness using UniProt.

### Immunoblotting

The samples were boiled in 5× Laemmli buffer for 5–10 min and run on 8% polyacrylamide gels at 140–150 V for 1 h. The proteins were transferred onto PVDF membranes (Sigma-Aldrich) by wet transfer at 100 V for 1 h. The membranes were then blocked with 5% nonfat milk in TBS containing 0.1% Tween 20 (TBST) for 1 h at RT. The membranes were washed in TBST and then incubated overnight at 4 °C in primary antibody diluted in blocking buffer (used at 1:1000–1:15000 dilution, dependent on antibody). The membranes were then rinsed extensively the following day in TBST and incubated in HRP-tagged secondary antibody in blocking buffer or TBST for 1 h at RT. Membranes were then developed using enhanced chemiluminescence (Thermo Fisher Scientific).

### Immunostaining

The cells were seeded and transfected using Lipofectamine 3000 (Thermo Fisher Scientific) on 18-mm glass cover slips (Thermo Fisher Scientific) coated with poly-l-lysine (Sigma-Aldrich) in 12-well plates at 80,000 cells/well. 48 h post-transfection, the cells were washed in prewarmed PBS and fixed with 4% PFA in PBS for 15 min at RT. The PFA was neutralized by washing the cover slips in 50 mm glycine in PBS for 10 min at RT. Fixed cells were permeabilized by 0.5% Triton X-100 in PBS for 5 min at RT. The cover slips were then washed in PBS, blocked in 5% BSA in PBS containing 0.05% Tween 20 (PBST) for 30 min at RT, and washed with PBST. Next, the cells were incubated in primary antibody (1:100 dilution) in PBST for 1 h at RT. The cover slips were washed in PBST and the cells were then incubated in fluorescently labeled secondary antibody in PBST for 1 h in the dark at RT. The cover slips were again washed in PBST to remove the unbound secondary antibody, dried, mounted on a glass slide with Prolong Diamond mounting medium containing 4′,6-diamidino-2-phenylindole (Thermo Fisher Scientific), and sealed with transparent nail polish. Stained cells were then imaged using Leica TCS SP5 confocal microscope under appropriate channels. The images were analyzed using LAS X software (Leica Microsystems) and merged in Adobe Photoshop. Quantification of inclusion bodies was done by manual counting.

### In vitro aggregation assay

At 24 h post-transfection, Huh7.5 CRT KO cells transfected with eYFP-ATZ encoding plasmids were semi-permeabilized in buffer containing 50 mm HEPES, pH 7.5, 150 mm NaCl, 2 mm CaCl_2_, and 0.01% digitonin supplemented with 10 mm *N*-ethylmaleimide and 1 mm PMSF on ice for 10 min. Next, the semi-permeabilized cells were centrifuged at high speed (16,250 × *g*) for 10 min. The supernatant fraction was discarded. The pellet fraction, enriched in membranes including the ER, was collected and subsequently lysed in RIPA buffer containing 0.1% SDS with 10 mm *N*-ethylmaleimide and 1 mm PMSF for 30 min at 4 °C with gentle agitation. The pellet-fraction lysate was again cleared by centrifugation at 16,250 × *g* for 10 min, and the remaining protein concentration of the clarified supernatant fraction was measured by BCA. The remaining supernatant fraction was further diluted 10-fold before being incubated with an equivalent amount (in micrograms) of purified recombinant human CRT (expressed and purified as described previously for mouse CRT (36)) or BSA or with one-half or one-quarter the lysate amounts of CRT, along with 100 μm ATP and 10 mm MgCl_2_. Each sample was then incubated on a magnetic stirrer at 37 °C for 30 min. The samples were subsequently centrifuged at 16,250 × *g* for 10 min to generate a supernatant fraction that contained soluble eYFP-ATZ and a new pellet fraction that harbored insoluble eYFP-ATZ. After reconstitution into equal volumes of Laemmli buffer and boiling, both fractions were subject to SDS-PAGE followed by immunoblotting.

### PNGase F and Endo H digestions

Huh7.5 CRT KO and WT cells were transfected with eYFP-ATZ–encoding plasmids or left untransfected and harvested at 24 h post-transfection. The cells were lysed in 1% Triton X-100 lysis buffer and cleared by centrifugation as described above. The cleared lysate concentrations were equalized based on BCA assays. PNGase F and Endo H digestion were done based on the New England BioLabs protocol: 2 μl of 10× Glycoprotein Denaturing Buffer was added to 40 μg of protein from each sample and samples were denatured by heating at 100 °C for 10 min. Denatured protein samples were then split in two. For PNGase F digestion, both samples were incubated with 2 μl of 10× GlycoBuffer 2, 2 μl of 10% Nonidet P-40, and either 1 μl of PNGase or 1 μl of deionized water (for the undigested controls). For Endo H digestion, both samples were incubated with 2 μl of 10× GlycoBuffer 3 and either 1.5 μl of Endo H or 1 μl of deionized water (for the undigested controls). All samples were then incubated at 37 °C for 1 h. After incubation, the samples were boiled in Laemmli buffer for immunoblotting.

### Statistical analysis

The majority of statistical analyses were performed with GraphPad Prism (version 7.0 or 8.0). One sample *t* tests for the MS data were performed with Microsoft Excel.

## Data availability

MS data have been deposited in ProteomeXchange and are available with identifier PXD020562. Project name: Calreticulin enhances the secretory trafficking of a polymerogenic α1-antitrypsin. The mass spectrometry proteomics data have been deposited to the Proteome Xchange Consortium via the PRIDE partner repository with the dataset identifier PXD020562.
