# Supplementary material for: Calreticulin enhances the secretory trafficking of a misfolded α-1-antitrypsin
Source: J Biol Chem. 2021 Jan 13;295(49):16754–72. doi: 10.1074/jbc.RA120.014372 (PMC7864070; doi:10.1074/jbc.RA120.014372)
Supplement: Supplementary file 1 [file mmc1.zip › 160863_3_supp_596467_qgkgmj.docx]

**SUPPORTING INFORMATION**

**Table S1.** Media to cell ratio calculations. In the attached file, there are four sheets corresponding to Figures 1, 3, 4, and S1. The sheets show the raw data and all relevant normalizations and calculations.

**Table S2.** Calculations for CRT ko/WT and CNX ko/WT abundance ratios of factors identified in MS data. In the attached file, there are fourteen sheets corresponding to Figures 6A, S6A, 6B, and S6B. The sheets show the raw data and all relevant normalizations and calculations of selected factors in our analyses.


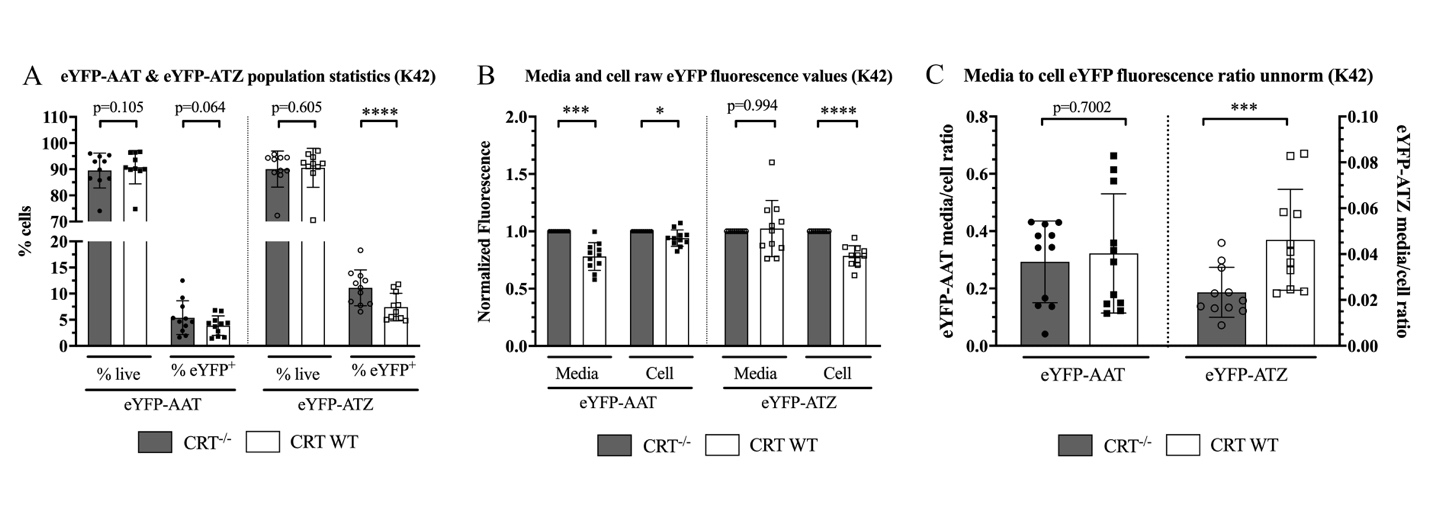


**Figure S1. CRT promotes the secretion of ATZ in K42 mouse embryonic fibroblasts**

**A)** Percentage live cells of all cells (pre-gated on forward and side scatter), and percentage of eYFP^+^ cells identified from the total live cell population. **B)** Total media fluorescence and cell MFI values were normalized relative to corresponding values from CRT^-/-^ cells in eYFP-AAT or eYFP-ATZ transfected cells. **C)** The ratio between the media and cell fluorescence values calculated as $\frac{Media Fluorescence}{Cellular eYFP MFI \times Number of eYFP+ cells}$ was obtained. Data were obtained from an additional 11 independent transfections of two independent sets of K42 CRT^-/-^ and CRT WT transductions over those shown in Figure 1 and are shown as mean ± SD. Paired two-tailed t-tests were performed in all cases. Since the data in B is normalized, those values were log-transformed prior to statistical analysis. * p <0.05, *** p ≤0.001, **** p <0.0001, compared to CRT^-/-^ condition.


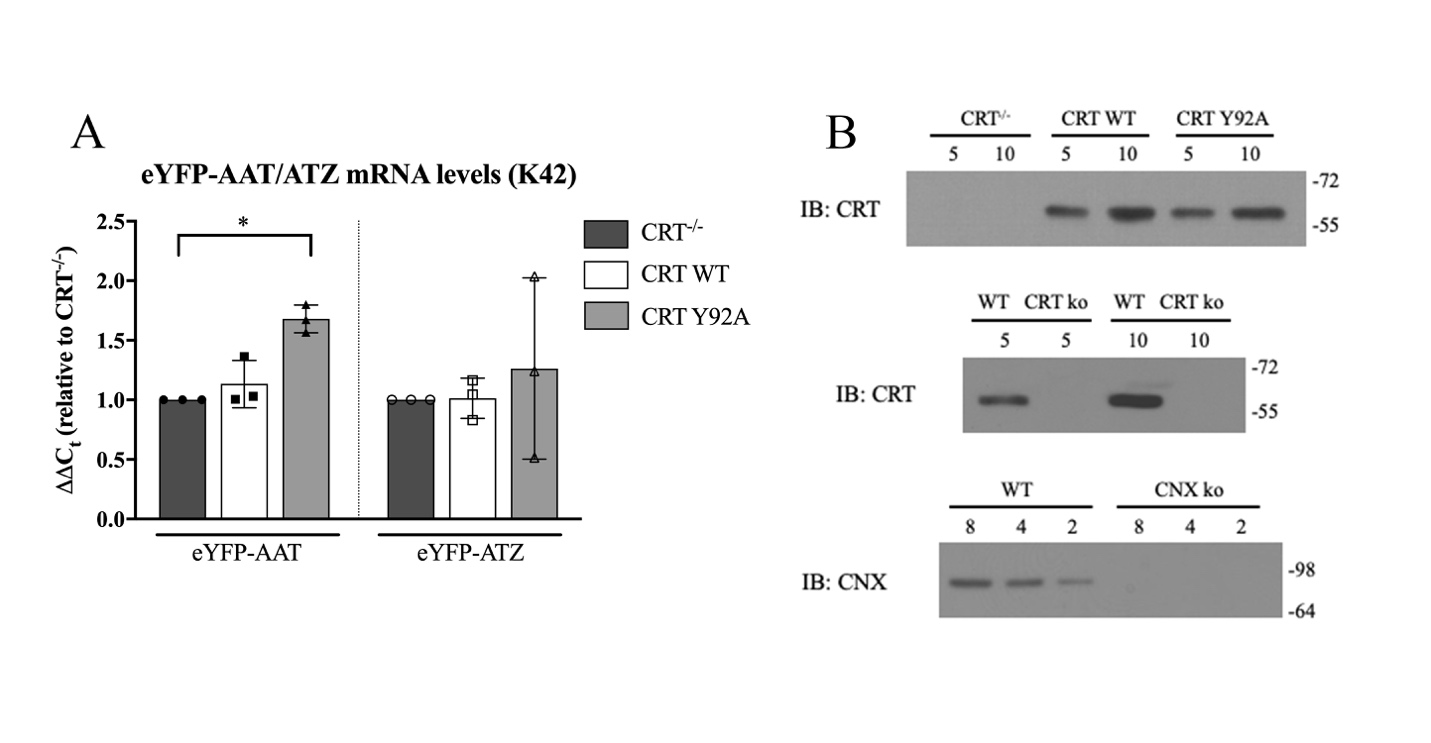


**Figure S2. eYFP-AAT and eYFP-ATZ expression in K42 MEFs, CRT reconstitution in K42 cells, and CRT and CNX knockout in Huh7.5 cells**

**A)** Quantitative RT-PCR was performed using primers against *SERPINA1* in K42 cells transduced with MSCV vector encoding CRT WT, CRT Y92A, or an empty control (CRT^-/-^) and transfected with either eYFP-AAT (*Left*) or eYFP-ATZ (*Right*). *Actb* and *Hprt* were used as housekeeping genes. Data were obtained from 3 replicates, and are shown as mean ± SD. CRT^-/-^ cells were used for normalizing ΔC_t_ values. RM one-way ANOVA was performed on log-transformed data. * p <0.05. **B)** (Top) K42 cells transduced with a retroviral vector encoding CRT WT, CRT Y92A, or an empty control (CRT^-/-^) were lysed and subjected to SDS-PAGE followed by immunoblotting against CRT. 5 and 10 micrograms of lysate from each cell type were loaded. (Middle) Huh7.5 cells transduced with an empty lentiviral vector or vector that silences CRT expression were lysed and subjected to SDS-PAGE followed by immunoblotting against CRT. 5 and 10 micrograms of lysate from cell type were loaded. (Bottom) Huh7.5 cells transduced with empty lentiviral vector or vector that silences CNX expression were lysed and subjected to SDS-PAGE followed by immunoblotting against CNX. 8, 4, and 2 micrograms of lysate from each cell type were loaded.

**
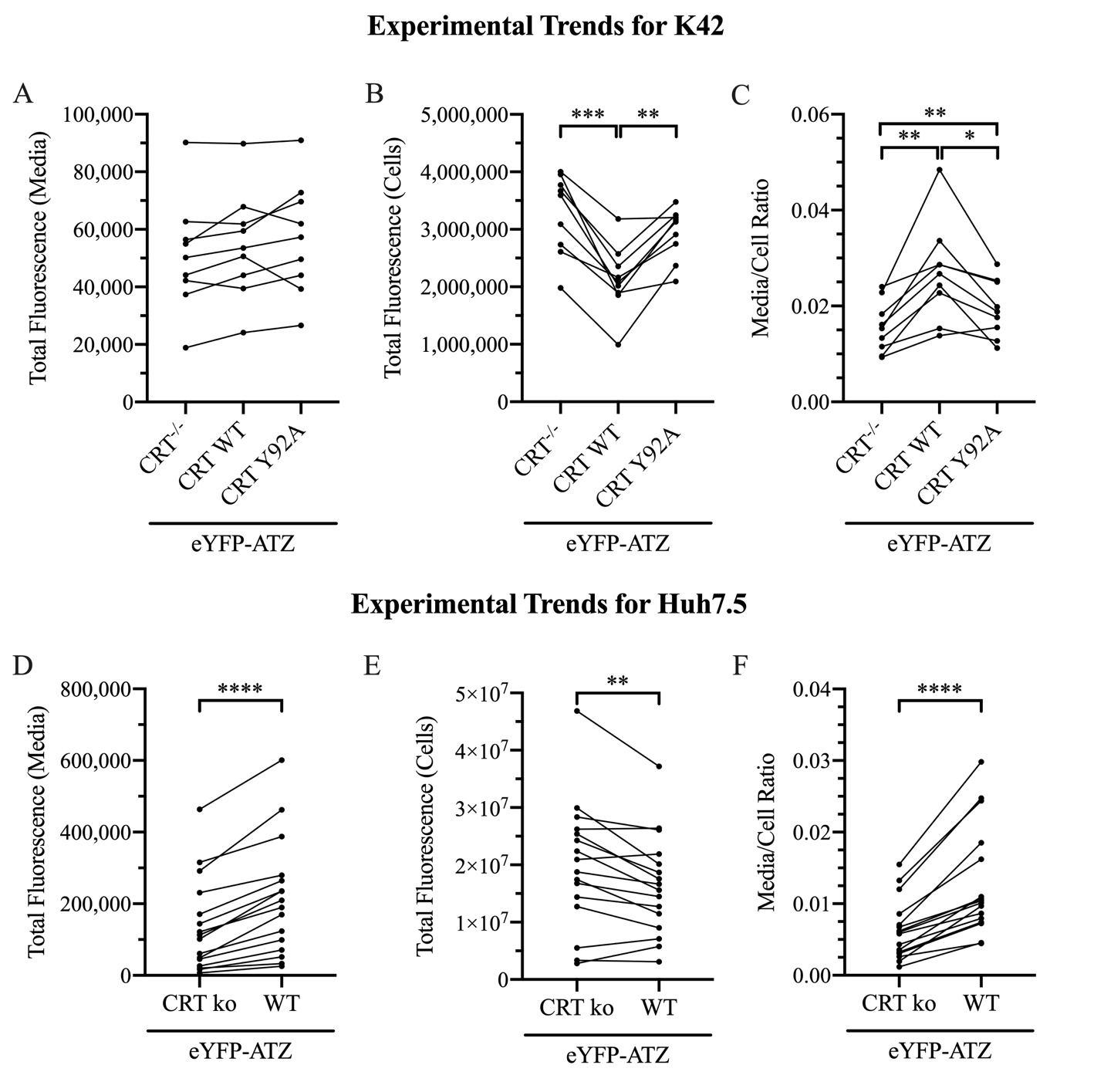
**

**Figure S3. eYFP-ATZ fluorescence in media and cells and media/cell eYFP-ATZ fluorescence ratios in individual experiments of transfected K42 and Huh7.5 cells expressing or lacking CRT**

Experiments of selected data from Figures 1 and 3 are replotted as line graphs, with values obtained from experiments performed on a given day connected by lines. **A and D)** Media fluorescence values were background subtracted using the corresponding untransfected controls, and the total fluorescence is plotted, corresponding to data in Figures 1E, right panel (A) or 3C (D). **B and E)** Total cellular fluorescence was calculated as: Cellular eYFP MFI × # of eYFP+ cells, for eYFP-ATZ transfected K42 (B) or Huh7.5 cells (D). **C and F)** Media/cell ratios for experiments shown in Figure 1F, right panel (C) or 3D, right, panel (F). RM one-way ANOVA analysis was performed for A-C and paired two-tailed t-tests were performed for D-F. * p <0.05, ** p <0.01, *** p<0.001. **D-F)**. ** p <0.01, **** p<0.0001.


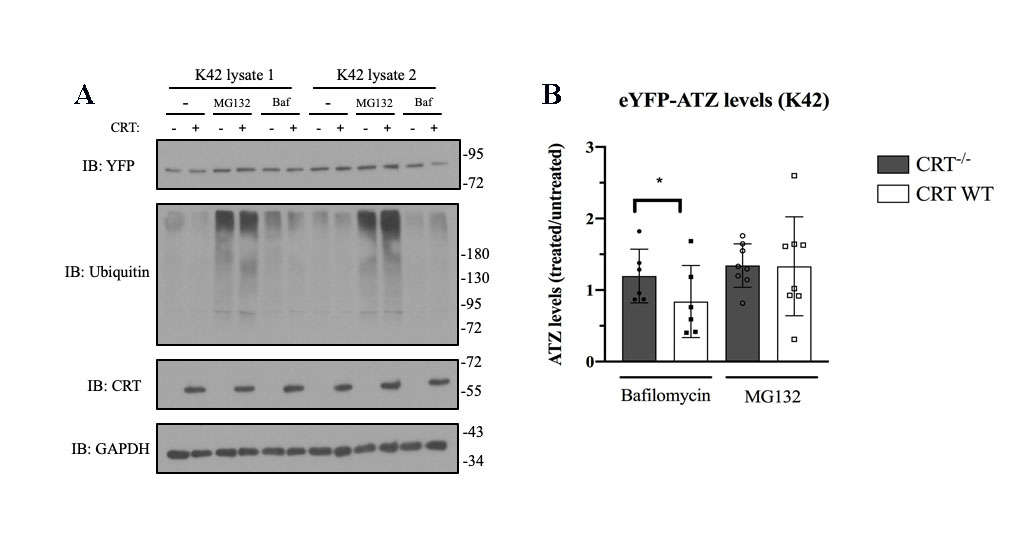


**Figure S4. The influences of CRT on ATZ degradation in K42 cells**

K42 cells were transfected with eYFP-ATZ encoding plasmids and treated with either 100 nM Bafilomycin or 10 μg/mL MG132 or left untreated at 20 h post transfection. At 24 h, cells were harvested and lysed in 1% Triton buffer. The lysates were subject to SDS-PAGE and immunoblotting for YFP, Ubiquitin, CRT, and GAPDH. **A)** Representative blots from two experiments (indicated as lysates 1 and 2 respectively) for indicated proteins in CRT WT or CRT^-/-^ K42 cells treated with Bafilomycin, MG132, or left untreated. **B)** Quantification of eYFP-ATZ levels normalized to GAPDH, then normalized to their respective untreated controls. Data quantified over at least 6 independent experiments are shown as mean ± SD. Paired two-tailed t-tests were performed comparing CRT^-/-^ and CRT WT.


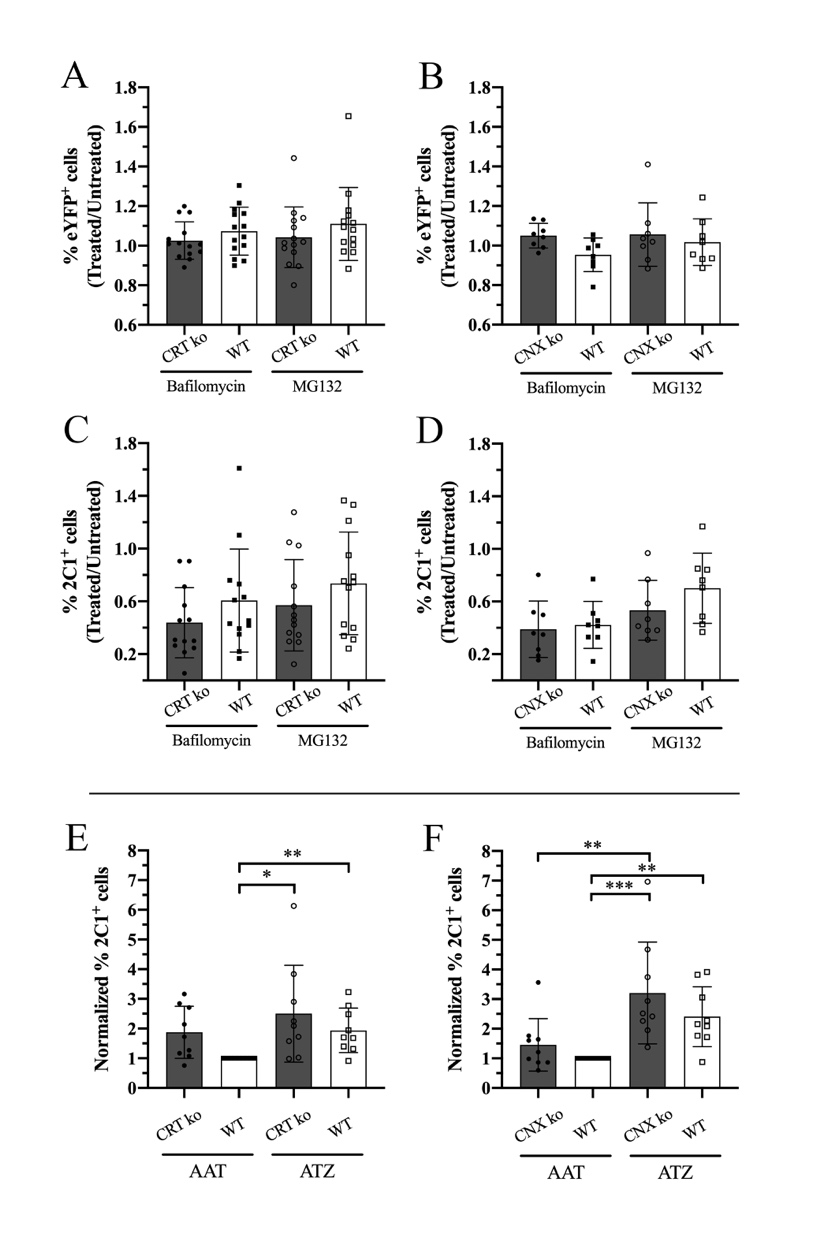


**Figure S5. Influences of CRT and CNX on polymeric ATZ accumulation and ATZ degradation - % eYFP^+^ and % 2C1^+^ cell populations**

**A-D)** Huh7.5 CRT ko, CNX ko, or corresponding WT cells as indicated were transfected with eYFP-ATZ-encoding plasmids and treated with 100 nM Bafilomycin or 10 μg/mL MG132 or left untreated at 20 h post-transfection. At 24 h post-transfection, cells were harvested, stained with 2C1, and analyzed. The polymeric ATZ (2C1) gate was determined by gating on forward and side scatter, live cells, then eYFP^+^ cells. The secondary antibody staining control was used as the cut-off for setting the polymeric ATZ gate. For each cell type and drug-treatment condition, ratios of signals from drug treated/untreated cells were measured to calculate % eYFP^+^ cell population ratios **(A and B)**, or % 2C1^+^ cell population ratios **(C and D)**. Data for CRT ko and WT were obtained from at least 13 independent replicates, 8 of which were conducted in parallel with CNX ko. Data for CNX ko and WT were obtained from 8 independent replicates. All data and are shown as mean ± SD. RM one-way ANOVA analysis was performed, and p-values are reported for comparisons of WT and ko conditions for each drug treatment. **E-F)** Huh7.5 CRT ko, CNX ko, or corresponding WT cells as indicated were transfected with eYFP-AAT or eYFP-ATZ encoding plasmids and stained with the polymer-selective antibody, 2C1, 48 h post-transfection. % 2C1^+^ cell populations (of eYFP^+^ populations) are shown (gated as described in A-D). Data quantified over 9 independent experiments are shown as mean ± SD. RM one-way ANOVA analysis was performed on log-transformed data in E and F. * p <0.05, ** p <0.01, *** p<0.001.

**
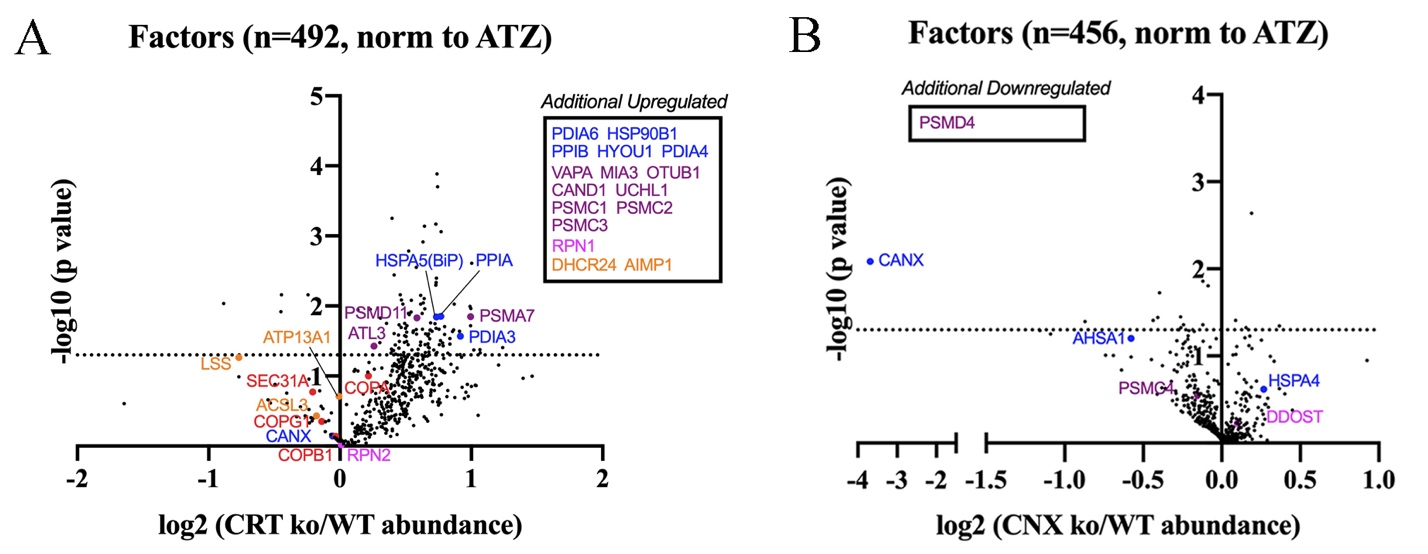
**

**Figure S6. eYFP-ATZ interactomes in Huh7.5 CRT ko and CNX ko cells, based on normalization to ATZ signals in WT vs. ko cells**

Volcano plots of the MS data obtained for Figure 6, but normalized relative to recovered eYFP-ATZ abundance in A) Huh7.5 CRT ko relative to the corresponding WT cells or B) Huh7.5 CNX ko and the corresponding WT cells. The ko/WT abundance ratios for each protein were calculated by dividing the specific PSM values of ko cells by their corresponding PSM values from WT cells and then normalizing relative to the eYFP-ATZ PSM ratio within each experiment: $\frac{(Factor \#PSMs in ko)/(Factor \#PSMs in WT)}{( eYFP-ATZ \#PSMs in ko)/(eYFP-ATZ \#PSMs in WT)}$. The dotted line indicates the significance threshold (p<0.05). Data shown were obtained and averaged from 3 independent experiments. P-values were calculated based on log-transformations of the normalized abundance ratios, followed by paired one sample t-tests of CRT ko and WT conditions for each factor. The same significant factors identified in Figure 6A and 6B are highlighted within each plot, and new ER-related factors with significantly higher or lower abundance ratios (p<0.05) in ko cells relative to WT cells are highlighted within the inset boxes. The same color scheme is used as described in Figure 6.


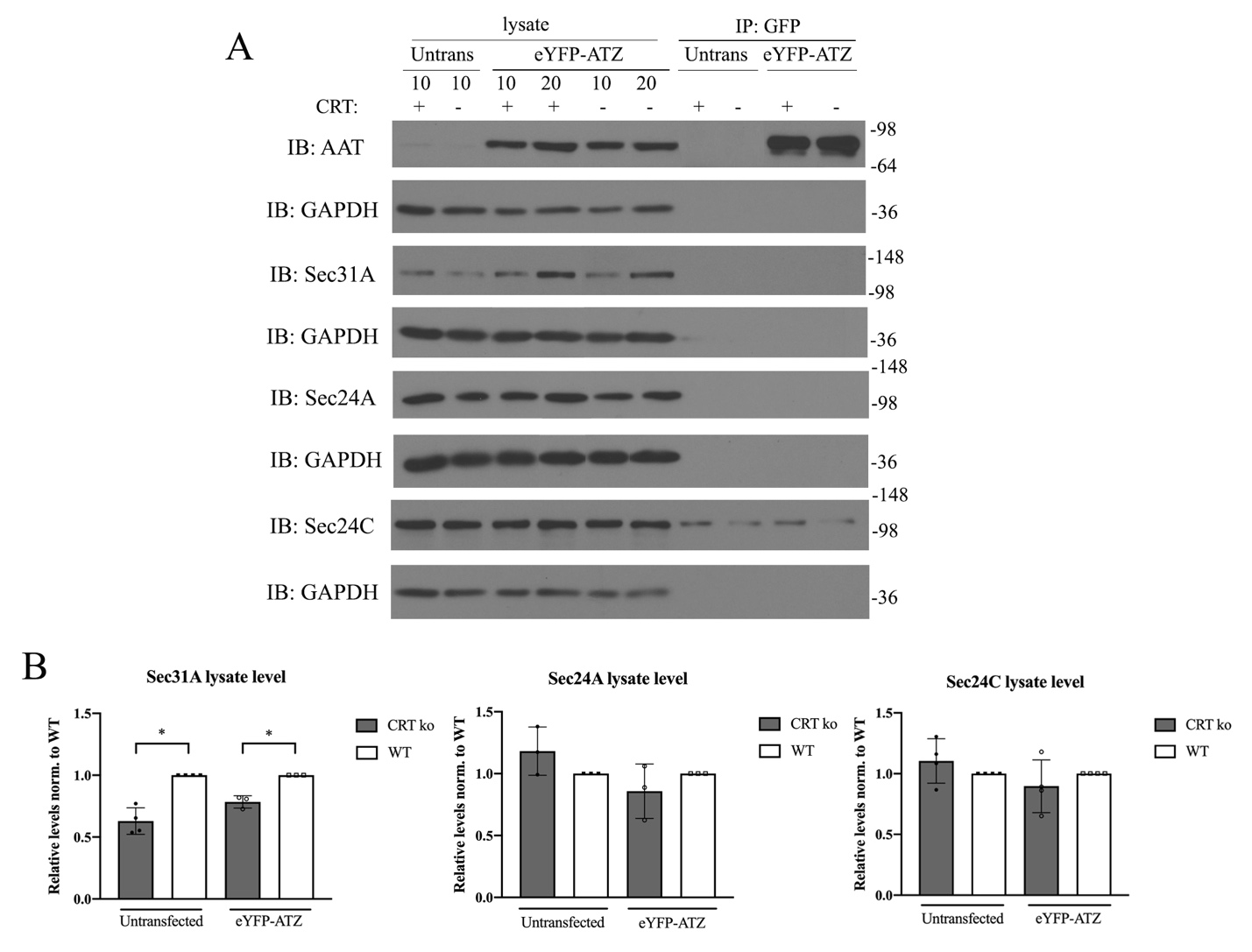


**Figure S7. CRT’s effect on COPII component expression**

**A)** Representative blots from anti-GFP co-immunoprecipitation experiments in Huh7.5 CRT ko and WT cells. eYFP-ATZ transfected cells were lysed and immunoprecipitated with anti-GFP beads as described for Figure 6, followed by immunoblotting for the indicated proteins. GAPDH was used as a loading control. **B)** Densitometric quantifications for Sec31A, Sec24A and Sec24C lysate levels in CRT ko and WT cells. Protein levels were calculated by dividing the band intensities for each Sec protein by the corresponding GAPDH intensities. Ratios were then normalized relative to WT cells. Data were obtained from at least 3 independent experiments and are shown as mean ± SD. Paired one-sample t-tests were performed on log-transformed, normalized data comparing CRT ko with WT intensities in untransfected and transfected cells. * p <0.05.
